# Mechanisms and integrative machine learning approaches to blood–brain barrier biomarker profiling for personalized ischemic stroke management

**DOI:** 10.14814/phy2.71003

**Published:** 2026-07-03

**Authors:** John Nzobokela, Lweendo Muchaili, Joy Kaluba Mangimela, Annet Kirabo, Sepiso K. Masenga

**Affiliations:** ^1^ Department of Pathology Ndola Teaching Hospital Ndola Zambia; ^2^ HAND Research Group, School of Medicine and Health Sciences Mulungushi University Livingstone Zambia; ^3^ Department of Cardiovascular Science and Metabolic Diseases Livingstone Center for Prevention and Translational Science Livingstone Zambia; ^4^ Department of Medicine Vanderbilt University Medical Center Nashville Tennessee USA; ^5^ Department of Molecular Physiology and Biophysics Vanderbilt University Medical Center Nashville Tennessee USA; ^6^ Vanderbilt Institute for Global Health Vanderbilt University Medical Center Nashville Tennessee USA; ^7^ Vanderbilt Center for Immunobiology Vanderbilt University Medical Center Nashville Tennessee USA; ^8^ Vanderbilt Institute for Infection, Immunology and Inflammation Vanderbilt University Medical Center Nashville Tennessee USA

**Keywords:** biomarkers, blood–brain barrier, deep learning, ischemic stroke, LASSO regression, machine learning, multi‐omics, neurovascular unit, oxidative stress, Support Vector Machine, vascular remodeling

## Abstract

Ischemic stroke remains a leading cause of death and disability worldwide, with blood–brain barrier (BBB) disruption playing a central role in vasogenic edema, neuroinflammation, hemorrhagic transformation, and secondary neuronal injury. The BBB is a specialized neurovascular unit composed of endothelial tight junctions, pericytes, astrocytes, and basement membrane structures that undergo coordinated molecular and cellular changes during ischemia–reperfusion injury, generating diverse biomarker signatures including endothelial dysfunction, oxidative stress, inflammatory mediators, and extracellular matrix remodeling. However, conventional biomarkers and imaging approaches fail to fully capture the dynamic and heterogeneous nature of BBB injury. Meaningful interpretation of BBB‐derived biomarkers requires mechanistic understanding of their molecular and cellular origins, making the integration of BBB pathophysiology with computational modeling essential for clinically relevant translation. Recent advances in machine learning (ML) and deep learning (DL) enable integration of neuroimaging, molecular, clinical, and multi‐omics data to characterize BBB dysfunction and improve prediction of stroke outcomes. ML‐based models have demonstrated value in identifying BBB‐related signatures associated with infarct progression, hemorrhagic transformation, and functional recovery, while deep neural networks enhance lesion segmentation and prognostic modeling. Despite this progress, challenges including data heterogeneity, limited longitudinal datasets, and model interpretability remain barriers to clinical translation. This review integrates the molecular and cellular mechanisms of BBB disruption with machine learning approaches for BBB biomarker profiling, highlighting a pathway toward biologically informed, personalized ischemic stroke management.

## 1 INTRODUCTION

More than five decades into the modern era of stroke research and management, the stroke continues to outpace medical innovations and impose a growing global health burden (Feigin et al., 2023). Despite advances in prevention and acute care, stroke remains a leading cause of death and disability, costing over US$890 billion annually (about 0.66% of global gross domestic product (GDP)) (Feigin et al., 2025). From 1990 to 2021, stroke incidence rose by 70%, deaths by 44%, and prevalence by 86%, with low‐ and middle‐income countries bearing nearly 87% of deaths and 89% of disability‐adjusted life‐years (DALYs) (Feigin et al., 2023; Feigin et al., 2025). Recently, machine learning technologies, particularly deep learning and neural networks, have emerged as powerful tools in stroke research, enabling precise image‐based detection, biomarker profiling, and data‐driven personalization of care, marking a shift toward truly intelligent and individualized stroke management (Kousar et al., 2025).

Understanding ischemic stroke at a mechanistic and predictive level requires linking molecular and cellular injury processes with measurable biomarkers and computational models. In this context, blood–brain barrier (BBB) disruption represents a central pathophysiological event that connects ischemic injury to downstream biomarker release and provides a mechanistic foundation for data‐driven prediction and precision modeling approaches.

The BBB is a specialized, highly selective interface that maintains central nervous system homeostasis by regulating the exchange of molecules between the blood and brain (Kadry et al., 2020). It consists of endothelial cells joined by tight junctions (occludin, claudin‐5, ZO‐1, and ZO‐2), supported by pericytes, astrocytic end‐feet, and a basement membrane that collectively preserve barrier integrity and protect neural tissue from toxins and pathogens (Chen et al., 2012; Fong et al., 2024). During ischemia–reperfusion injury, oxygen and glucose deprivation followed by oxidative and inflammatory stress disrupt these structural components, leading to degradation of tight junction proteins, endothelial dysfunction, cytokine release such as tumor necrosis factor‐alpha (TNF‐α), interleukin (IL)‐1β, and IL‐6, and vasogenic edema (Guo et al., 2023). This breakdown of the BBB exacerbates neuroinflammation, edema, and neuronal injury, underscoring its pivotal role in stroke pathophysiology and its potential as a target for biomarker discovery and advanced computational modeling (Kousar et al., 2025).

The critical role of the BBB in ischemic stroke highlights the need for reliable biomarkers to assess barrier integrity and guide stroke management. While traditional markers such as radiolabeled compounds, dextrans, and cerebrospinal fluid (CSF) proteins and imaging techniques such as dynamic contrast‐enhanced magnetic resonance imaging (DCE‐MRI), dynamic susceptibility contrast magnetic resonance imaging (DSC‐MRI), and arterial spin labeling (ASL) provide valuable insights, no single approach fully captures the dynamic and complex nature of BBB disruption (French et al., 2025; Kadry et al., 2020).

Blood‐based biomarkers hold promise for assessing BBB integrity but often suffer from variability and low sensitivity. Recent studies have shown that while certain biomarkers like neuron‐specific enolase and glial fibrillary acidic protein are elevated in acute ischemic stroke (AIS) patients, their diagnostic performance varies significantly across studies (Rahmig et al., 2024). These challenges underscore the potential of computational and machine learning approaches, which can integrate multi‐modal data, including imaging, molecular, and peripheral blood profiles, to identify patterns, predict outcomes, and support personalized ischemic stroke management (Kousar et al., 2025; Liu et al., 2025). This review integrates molecular and cellular mechanisms of BBB disruption with machine learning approaches for BBB biomarker profiling, highlighting how mechanistically informed computational modeling can advance personalized prediction, diagnosis, and therapeutic strategies in ischemic stroke.

## 2 PATHOPHYSIOLOGY AND TEMPORAL DYNAMICS OF BLOOD–BRAIN BARRIER DYSFUNCTION IN ISCHEMIC STROKE

### 2.1 The blood–brain barrier: Structure and function in neurovascular homeostasis

The BBB is a highly specialized interface that maintains CNS homeostasis through coordinated molecular, cellular, and signaling mechanisms (Abdullahi et al., 2018; Masenga & Kirabo, 2024). It is composed of brain microvascular endothelial cells interconnected by tight junction proteins, such as claudin‐5, occludin, and VE‐cadherin, and supported by pericytes, astrocytic end‐feet, and the basement membrane, collectively forming a part of the neurovascular unit (NVU) (Candelario‐Jalil et al., 2022). The BBB controls the movement of molecules in both directions through specific carriers like glucose transporter‐1 and large neutral amino acid transporters. At the same time, efflux pumps such as P‐glycoprotein prevent foreign substances from entering (Hosoya et al., 2002). Immune surveillance is tightly controlled by low expression of adhesion molecules and regulated cytokine signaling.

The BBB also functions as a dynamic signaling hub, where interactions among endothelial cells, astrocytes, and pericytes modulate vascular permeability and metabolic coupling (Nian et al., 2020). Endothelial mechanotransduction plays a crucial role in this process; the endothelial glycocalyx, a glycoprotein–proteoglycan layer extending into the vessel lumen, is abundantly expressed on BBB endothelial cells and has been shown to regulate barrier permeability (Nian et al., 2020). Under physiological stress, redox‐sensitive pathways such as Nuclear factor erythroid 2‐related factor 2 (Nrf2) preserve mitochondrial integrity, whereas vascular endothelial growth factor (VEGF) and matrix metalloproteinases (MMP‐2 and MMP‐9) influence junctional stability and extracellular matrix remodeling (Abdullahi et al., 2018; Mathias et al., 2024). In ischemic stroke, dysregulation of these pathways leads to oxidative damage, tight junction degradation, and inflammatory infiltration, ultimately compromising BBB integrity (Abdullahi et al., 2018; Candelario‐Jalil et al., 2022).

### 2.2 Endothelial glycocalyx shedding (early hyperacute injury phase)

Endothelial glycocalyx shedding is a critical early event in ischemic stroke that contributes to BBB disruption and subsequent neuroinflammation (Balistreri et al., 2024). During ischemia, oxygen and glucose deprivation lead to oxidative stress, acidosis, and enzymatic activation particularly of C and hyaluronidase which degrade heparan sulfate and chondroitin sulfate components of the glycocalyx. This enzymatic breakdown compromises the vascular barrier, facilitating leukocyte infiltration and edema formation. Studies using mouse models of photothrombotic stroke demonstrate that glycocalyx degradation is accompanied by elevated heparanase activity and changes in ionic composition of the cerebrovascular glycocalyx, underscoring its dynamic remodeling during the acute phase of stroke (Zhu et al., 2018).

Rather than occurring globally, glycocalyx shedding appears spatially heterogeneous, with vascular regions exposed to mechanical stress or oxidative imbalance being particularly susceptible (Jin et al., 2021; Shi et al., 2025). Within the brain, this localized degradation carries disproportionately severe consequences, as even subtle BBB compromise disrupts neurovascular unit homeostasis and accelerates ischemic injury progression (Zhu et al., 2022).

At the molecular level, endothelial glycocalyx degradation is amplified by reactive aldehydes such as acrolein, a toxic byproduct of lipid peroxidation generated during ischemia–reperfusion injury (Ćurko‐Cofek et al., 2024). Acrolein activates pro‐heparanase through post‐translational modification of specific lysine residues (Lys107, Lys139, and Lys161) located near the 6‐kDa linker region, thereby converting the inactive proenzyme into its catalytically active form (Ko et al., 2021). In cerebral vessels, pro‐heparanase expression markedly increases after stroke onset, and co‐administration of N‐acetylcysteine and glycosaminoglycan oligosaccharides significantly reduces infarct volume compared with N‐acetylcysteine alone, indicating that oxidative activation of pro‐heparanase contributes directly to glycocalyx degradation and cerebrovascular injury (Ko et al., 2021). Because pro‐heparanase but not mature pro‐heparanase associates extracellularly with heparan sulfate proteoglycans, acrolein‐modified pro‐heparanase represents a critical extracellular effector of glycocalyx loss and a promising therapeutic target for endothelial protection (Ko et al., 2021).

**FIGURE 1:**
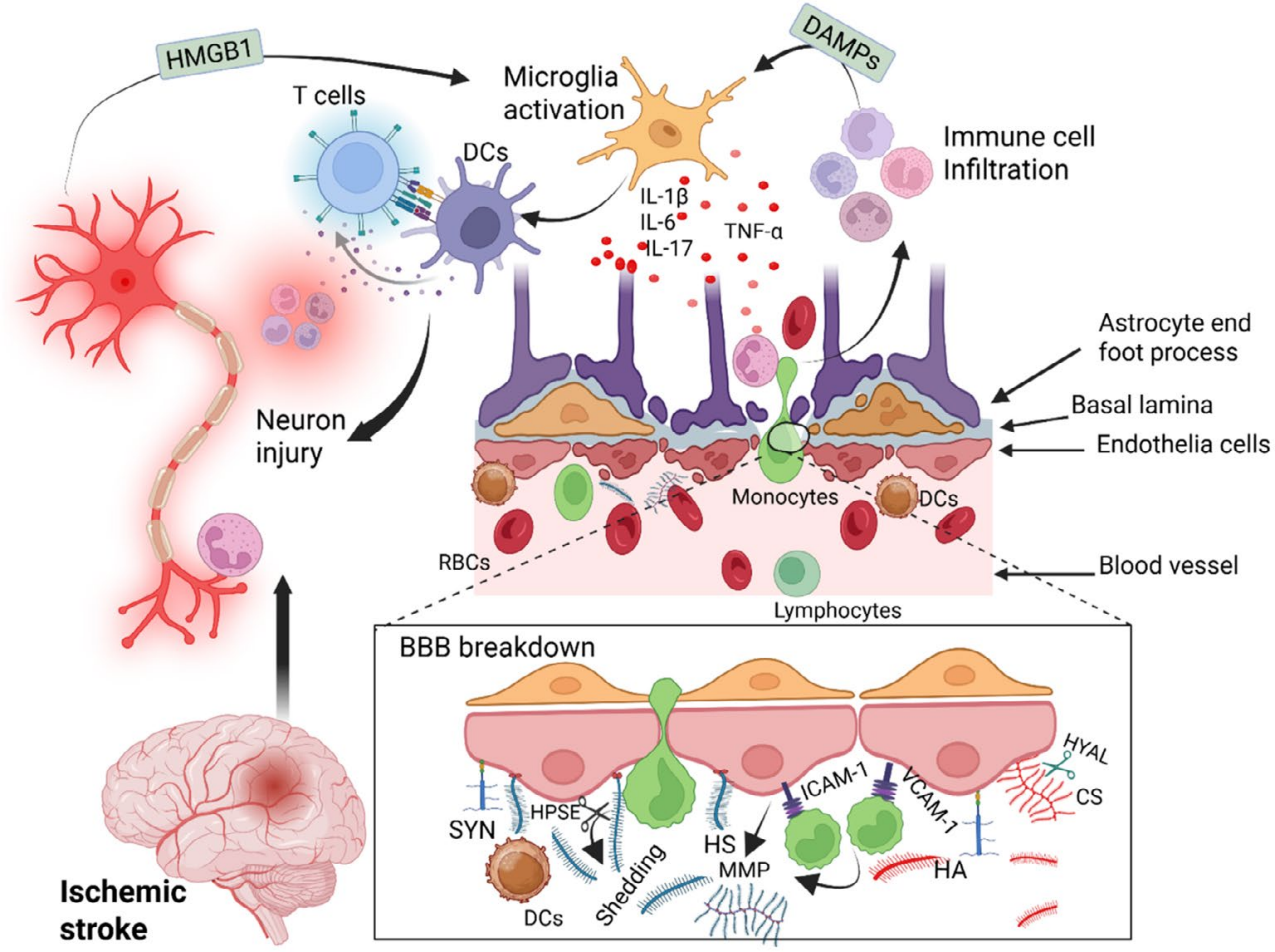
Endothelial glycocalyx shedding as a central mechanism in blood brain barrier breakdown and neuroinflammation after ischemic stroke. Ischemia–reperfusion generates ROS and reactive aldehydes (e.g., acrolein), activating HPSE. Activated HPSE, together with HYAL and MMP, degrades glycocalyx components HS and CS, leading to endothelial barrier disruption and exposure of ICAM‐1 and VCAM‐1, thereby promoting leukocyte adhesion and neuroinflammation. Ischemic injury induces the release of DAMPs and HMGB1, activating microglia and infiltrating immune cells. Pro‐inflammatory cytokines amplify neurovascular inflammation, while glycocalyx loss facilitates leukocyte transmigration across the BBB, disrupting astrocytic end‐feet and basal lamina, ultimately aggravating vascular leakage and secondary neuronal injury. BBB, blood–brain barrier; CS, chondroitin sulfate; DAMPs, damage‐associated molecular patterns; DCs, dendritic cells; HA, hyaluronan; HMGB1, high‐mobility group box 1; HPSE, pro‐heparanase; HYAL, hyaluronidase; ICAM‐1, intercellular adhesion molecule‐1; IL‐17, interleukin‐17; IL‐1β, interleukin‐1 beta; IL‐6, interleukin‐6; MMP, matrix metalloproteinase; SYN, snydecan‐1; TNF‐α, tumor necrosis factor‐alpha; VCAM‐1, vascular cell adhesion molecule‐1.

This aldehyde‐driven pathway links oxidative stress and mechanotransduction failure to glycocalyx degradation and BBB leakage, amplifying vascular vulnerability (Nian et al., 2020). The ensuing exposure of endothelial adhesion molecules such as intercellular adhesion molecule‐1 (ICAM‐1) and vascular cell adhesion molecule‐1 (VCAM‐1) promotes leukocyte recruitment, propagating neuroinflammation and secondary neuronal injury (Figure 1) (Dancy et al., 2024). Recent evidence of inhibition of heparanase or MMP‐9 has been shown to mitigate inflammatory markers and improve behavioral outcomes in experimental stroke, highlighting the therapeutic potential of preserving glycocalyx integrity (Nian et al., 2020; Zhu et al., 2018).

Recent multi‐modal studies integrating transcriptomics and advanced imaging have revealed that glycocalyx remodeling in ischemic stroke is bidirectional and cell‐type specific, affecting both endothelial and leukocyte surfaces (Arbaizar‐Rovirosa et al., 2023; Hansen et al., 2025). This evidence suggests that disruption of the endothelial glycocalyx represents not merely a structural deterioration but a mechanistic nexus linking vascular inflammation, BBB dysfunction, and impaired neurological recovery (Hansen et al., 2025). Machine learning (ML) and computational modeling have been applied to predict BBB permeability using multi‐modal data, demonstrating the feasibility of modeling BBB integrity and barrier disruption features (Jafarpour et al., 2024; Liu et al., 2021). Future efforts integrating molecular, imaging, and biomechanical data through machine learning frameworks could enable precise characterization of glycocalyx dynamics and identification of novel therapeutic targets. Protecting the glycocalyx whether by inhibiting enzymatic degradation, mitigating oxidative stress, or restoring biosynthetic pathways, thus remains a promising strategy to preserve BBB function and improve outcomes after ischemic stroke (Dancy et al., 2024; Ko et al., 2021; O'Hare et al., 2024). This early glycocalyx disruption represents the first measurable structural breakdown of BBB integrity, initiating the cascade of downstream oxidative and inflammatory injury that defines ischemic progression.

### 2.3 Oxidative stress and mitochondrial injury (propagation phase of BBB damage)

Following endothelial glycocalyx degradation and inflammatory activation, oxidative stress becomes a central mediator of neurovascular injury, driving mitochondrial dysfunction and amplifying blood–brain barrier breakdown (Candelario‐Jalil et al., 2022). Mitochondrial failure impairs oxidative phosphorylation, resulting in the accumulation of reactive oxygen species (ROS), endoplasmic reticulum stress, and disruption of ion homeostasis via Na^+^/K^+^ ATPase failure and Ca^2+^ overload. Elevated Ca^2+^ activates protein kinase C, which enhances nicotinamide adenine dinucleotide phosphate (NADPH) oxidase‐mediated ROS production (Abdullahi et al., 2018; Masenga & Kirabo, 2024). Excess ROS induce lipid peroxidation and oxidative damage to proteins, DNA, and RNA, culminating in neuronal dysfunction and death (Tian et al., 2024).

After ischemic stroke, two hallmark features of ferroptosis—lipid peroxidation and iron accumulation—are evident, accompanied by altered expression of ferroptosis‐related genes such as GPX4, ACSL4, and SLC7A11 (Hu et al., 2024). These processes are further modulated by mitochondrial oxidative stress through the nuclear factor erythroid 2‐related factor 2 (NRF2)‐antioxidant response element pathway (Hu et al., 2024; Tian et al., 2024). Upon activation, NRF2 triggers the transcription of a cascade of antioxidant and cytoprotective genes (Wang et al., 2022). The combined effects of BBB disruption, excitotoxicity, and inflammation disrupt iron metabolism and weaken the brain's antioxidant defense system, thereby amplifying oxidative imbalance and ferroptotic neuronal death. ROS activate nuclear factor kappa‐light‐chain‐enhancer of activated B cells (NF‐κB) signaling and the NLR family pyrin domain containing 3 (NLRP3) inflammasome, leading to transcription and secretion of pro‐inflammatory cytokines such as IL‐1β and IL‐18 (Masenga & Kirabo, 2024). Necrotic neuronal death further amplifies local inflammation, recruiting immune cells that exacerbate BBB damage.

These mechanistic events in ischemic stroke generate detectable molecular biomarkers that reflect the intensity of oxidative and inflammatory injury. Mitochondrial stress and deoxyribonucleic acid (DNA) damage are captured by Cyclin‐Dependent Kinase Inhibitor 1A (CDKN1A), which regulates cell cycle progression and DNA repair, and by antioxidant enzymes such as Glutathione Peroxidase 4 (GPX4), Peroxiredoxin 1 (PRDX1), and Peroxiredoxin 6 (PRDX6), which mitigate oxidative stress and lipid peroxidation (Pawluk et al., 2024; Zhang et al., 2024). Activation of redox‐sensitive pathways further produces transcriptional and protein‐level signatures measurable through RNA sequencing or targeted assays (Zhang et al., 2024).

Recent studies have demonstrated the integration of these biomarkers into machine learning models. Using transcriptomic data, Zhang et al. applied differential expression analysis, WGCNA, PPI network screening, and machine learning classifiers (Support Vector Machine and Random Forest) with feature intersection to identify CDKN1A, GPX4, PRDX1, and PRDX6 as key oxidative stress–ferroptosis biomarkers associated with ischemic stroke outcomes (Zhang et al., 2024). Weighted Gene Co‐expression Network Analysis and single‐cell RNA sequencing (scRNA‐seq) combined with machine learning further validated their relevance in oxidative stress and immune response pathways, highlighting their potential for patient stratification and early trajectory prediction (Li, Kang, et al., 2025; Zhao et al., 2024). These oxidative and mitochondrial alterations propagate and amplify the initial glycocalyx‐mediated BBB disruption, linking early structural injury to widespread cellular dysfunction.

### 2.4 Inflammatory signaling and neurovascular unit crosstalk

**FIGURE 2:**
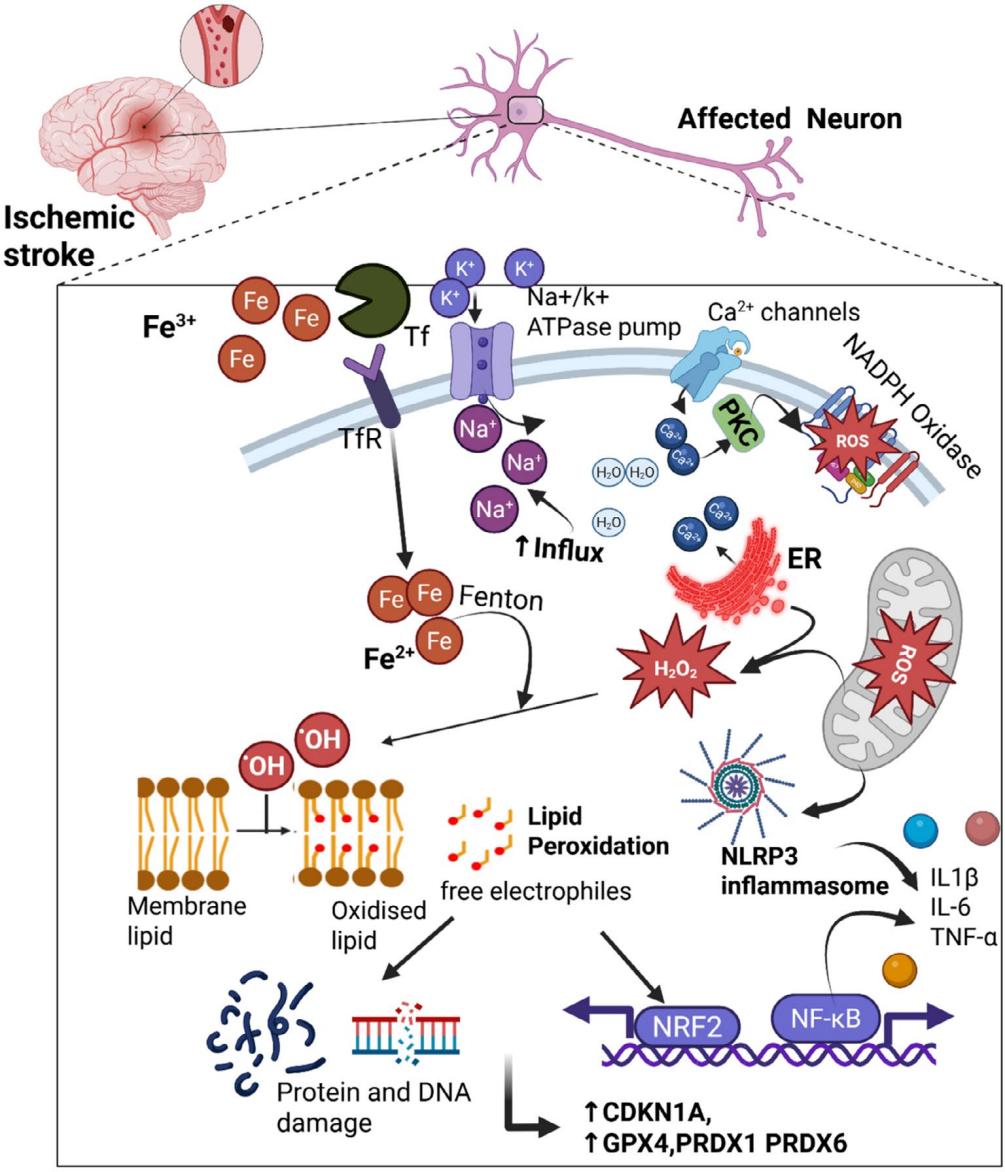
Oxidative stress, mitochondrial dysfunction, and ferroptosis following ischemic stroke. Ischemic stroke initiates mitochondrial dysfunction and oxidative stress, disrupting ionic homeostasis and promoting ferroptosis in neurons. Oxygen and glucose deprivation impair mitochondrial oxidative phosphorylation, increasing ROS and H_2_O_2_ production. Elevated intracellular calcium (Ca^2+^) via voltage‐gated channels and PKC activation increases NADPH oxidase activity, increasing ROS generation. Iron dysregulation occurs through TfR‐mediated uptake and Fenton reactions, producing •OH that drive lipid peroxidation and membrane damage. Accumulated oxidized lipids and electrophiles trigger ferroptosis and the activation of the NLRP3 inflammasome. This leads to the release of pro‐inflammatory cytokines such as IL‐1β, IL‐6, and TNF‐α. NRF2 and NF‐κB signaling pathways modulate the expression of antioxidant and stress‐response genes, including CDKN1A, GPX4, PRDX1, and PRDX6, linking oxidative injury to neuroinflammation and neuronal death. CDKN1A, cyclin‐dependent kinase inhibitor 1A; ER, endoplasmic reticulum; Fe, iron; GPX4, glutathione peroxidase 4; IL‐1β, interleukin‐1 beta; IL‐6, interleukin‐6; NADPH, nicotinamide adenine dinucleotide phosphate; NF‐κB, nuclear factor kappa‐light‐chain‐enhancer of activated B cells; NLRP3, NLR family pyrin domain‐containing 3; NRF2, nuclear factor erythroid 2–related factor 2; PKC, protein kinase C; PRDX1, peroxiredoxin 1; PRDX6, peroxiredoxin 6; ROS, reactive oxygen species; Tf, transferrin; TfR, transferrin receptor; TNF‐α, tumor necrosis factor‐alpha.

Ischemic stroke induces oxidative and mitochondrial injury that disrupts the NVU through coordinated cellular and molecular responses culminating in BBB dysfunction (Candelario‐Jalil et al., 2022). Activated microglia release proinflammatory cytokines such as IL‐1β and TNF‐α, promoting MMP‐9 upregulation and endothelial permeability (Candelario‐Jalil et al., 2022; Mayer & Fischer, 2024). Astrocytic metabolic failure, marked by impaired lactate shuttling and ionic imbalance, aggravates neuronal injury, while pericyte detachment destabilizes the microvasculature and predisposes to hemorrhagic transformation (Pacinella et al., 2025). Dying neurons release danger‐associated molecular patterns (DAMPs) such as ATP and high‐mobility group box 1, which activate pattern recognition receptors (Toll‐like receptors, purinergic receptor P2X7) and the NLRP3 inflammasome, amplifying cytokine cascades involving IL‐1β, IL‐6, IL‐17, and TNF‐α (Gülke et al., 2018; Masenga & Kirabo, 2024). Microglia and infiltrating macrophages, neutrophils, and γδ T cells drive sterile neuroinflammation, whereas regulatory T cells and transforming growth factor beta 1 (TGF‐β1) mediate later tissue repair (Candelario‐Jalil et al., 2022). Concurrent oxidative and nitrosative stress impair mitochondrial respiration and endothelial integrity, reinforcing BBB leakage and leukocyte transmigration via the upregulation of adhesion molecules (Figure 2) (Masenga & Kirabo, 2024). This inflammatory phase amplifies earlier oxidative and endothelial injury, converting localized BBB disruption into a systemic neurovascular inflammatory cascade.

Multicellular processes including microglial activation, astrocytic stress, pericyte dropout, endothelial dysfunction, and immune cell infiltration collectively link redox imbalance and cytokine signaling to BBB breakdown (Geranmayeh et al., 2019; Knox et al., 2022). Such molecular perturbations can be quantified using high‐throughput cytokine panels, transcriptomic profiling such as endothelial gene signatures after ischemia, and plasma proteomics in acute stroke (Angerfors et al., 2023; Arbaizar‐Rovirosa et al., 2023).

Machine learning algorithms integrate these datasets to identify key biomarkers and support personalized stroke prediction. For example, cytokine profiles combined with clinical laboratory and demographic data have been used to train Random Forest, Support Vector Machine, and Gradient Boosting models in large stroke cohorts. Feature selection and importance ranking within the Random Forest model highlighted IL‐6, IL‐5, IL‐10, and IL‐2 as top predictors, with the best‐performing model achieving an AUC of 0.74 in validation testing, demonstrating moderate generalization ability (Zhi et al., 2024). These ML‐driven frameworks enable biomarker prioritization and patient stratification, supporting an omics‐informed, ML guided approach to BBB profiling and personalized neurovascular protection.

### 2.5 Vascular remodeling and endothelial senescence (chronic phase of BBB dysfunction)

In the chronic phase of ischemic stroke, sustained oxidative stress, inflammation, and NVU disruption induce maladaptive vascular remodeling and endothelial senescence, which together perpetuate BBB leakiness and impair long‐term cerebrovascular integrity (Real et al., 2024; Venkat et al., 2017). Endothelial cells exposed to post‐ischemic stress exhibit diminished proliferative capacity and impaired tight‐junction restoration, while dysregulated extracellular matrix turnover mediated by MMP‐9 and its inhibitor tissue inhibitor of metalloproteinases‐1 (TIMP‐1) leads to vascular stiffness and fibrosis (Picos et al., 2025; Turner & Sharp, 2016). A recent prospective observational study demonstrated persistent upregulation of MMP‐9 and VEGF during the subacute and recovery stages of ischemic stroke, correlating with neurocognitive outcomes and vascular remodeling (Włodarczyk et al., 2024).

Senescence signaling is a key driver of chronic‐phase dysfunction following ischemic stroke (Huang, Xu, et al., 2024). Recent spatio‐temporal analyses in experimental models provide direct evidence of cellular senescence in the post‐ischemic brain. In male Wistar rats subjected to transient middle cerebral artery occlusion, neurons and microglia/macrophages in peri‐infarct regions exhibited significant increases in p16 and p21 expression, accumulation of senescence‐associated β‐galactosidase (SA‐β‐gal), and early induction of senescence‐associated secretory phenotype (SASP) markers, including IL‐6, IL‐1β, and TNF, as early as 24 h post‐reperfusion (Baixauli‐Martín et al., 2025). This chronic remodeling phase represents the long‐term consequence of unresolved early BBB injury and inflammatory amplification.

These senescence‐associated alterations provide a mechanistic basis for subsequent neurovascular and immune changes observed after stroke. Furthermore, DNA damage and nuclear stress markers, including Chk1, Chk2, and Lamin B1, remain altered up to 14 days post‐insult, confirming persistent senescence during the recovery phase (Baixauli‐Martín et al., 2025). Therefore, these mechanisms link endothelial and glial senescence to impaired BBB repair and chronic neuroinflammation, establishing cellular senescence as a central contributor to long‐term cerebrovascular dysfunction and post‐stroke cognitive decline.

Telomere attrition further contributes to vascular aging and impaired recovery. A recent study showed that individuals with shorter leukocyte telomeres (≤5.5 kb) exhibited nearly a three‐fold higher risk of ischemic stroke, emphasizing telomere length as a biomarker of biological vascular aging (Yetim et al., 2021). Supporting this, Gao et al. (2024) showed that endothelial‐specific telomerase inactivation in murine models accelerated vascular senescence and impaired BBB recovery, while Liang et al. (2025) confirmed that telomere erosion and methylation‐based biological aging indices predict worse long‐term outcomes (Gao et al., 2024; Liang et al., 2025). Thus, MMP‐9, p16^Ink4a^ and p21^Cip1/Waf1^and telomere shortening represent robust chronic‐phase biomarkers indicative of maladaptive vascular remodeling and senescence‐driven BBB dysfunction. These chronic biomarkers reflect the culmination of the earlier hyperacute, oxidative, and inflammatory phases of BBB disruption.

Integrating these longitudinal biomarkers into time‐series and survival machine learning models such as recurrent neural networks and Cox proportional hazards‐based algorithm enhances prediction of BBB recovery trajectories, recurrent stroke risk, and cognitive decline. Time‐series models capture dynamic fluctuations in MMP‐9 or p16^Ink4a^, while survival‐based frameworks connect senescence and telomere indices to long‐term vascular outcomes (Han & Kim, 2023). This systems‐level approach bridges molecular senescence mechanisms with clinical progression, laying the groundwork for personalized prognostic and therapeutic monitoring in ischemic stroke.

### 2.6 Temporal phases and predictive dynamics

BBB dysfunction in ischemic stroke evolves as a dynamic continuum across hyperacute, acute, subacute, and chronic phases, with distinct pathological features and biomarker signatures (Gao et al., 2023). This progression reflects a temporally organized cascade of vascular injury and repair processes rather than a static breakdown of barrier integrity.

In the hyperacute phase (less than 6 h post‐stroke), ischemia triggers immediate endothelial energy failure, cytoskeletal rearrangement, and tight junction disruption, resulting in early BBB leakage (Abdullahi et al., 2018). Oxidative stress and mitochondrial injury dominate, with biomarkers such as CDKN1A, GPX4, PRDX1, and PRDX6 reflecting reactive oxygen species induced cellular damage (Liang et al., 2025). LASSO regression and Random Forest models can stratify patients based on early barrier disruption (Emmerich et al., 2025; Pawluk et al., 2024). Intravital two‐photon imaging in mouse models demonstrates that BBB breach occurs along the arteriovenous axis within minutes, supporting rapid phase‐specific vascular responses (Protzmann et al., 2024).

During the acute phase (6–72 h post‐stroke), BBB permeability peaks (Bernardo‐Castro et al., 2020). Activated microglia release cytokines (IL‐1β and TNF‐α), upregulate MMP‐9, and drive endothelial injury, while astrocytic metabolic failure and pericyte detachment exacerbate barrier dysfunction (Emmerich et al., 2025; Mathias et al., 2024). These dynamic inflammatory and molecular changes can be captured using recurrent neural networks, gradient boosting machines, and deep learning autoencoders to model complex temporal interactions in multi‐modal datasets (Al‐Jehani et al., 2025; Li et al., 2023).

In the subacute phase (72 h to 6 weeks), partial BBB restoration begins as tight junctions reassemble, endothelial cells recover, and inflammation shifts toward reparative phenotypes. Longitudinal imaging studies using contrast‐enhanced MRI confirm gradual normalization of BBB permeability, while plasma MMP‐9/TIMP‐1 ratios, endothelial exosomal miRNAs, and epigenetic modifications (e.g., DNA methylation of claudin‐5 and Rho GTPase pathways) track ongoing repair and remodeling (Müller et al., 2021; Phillips et al., 2023; Włodarczyk et al., 2024). Multi‐modal machine learning models integrating these markers predict recovery trajectories and functional outcomes.

**FIGURE 3:**
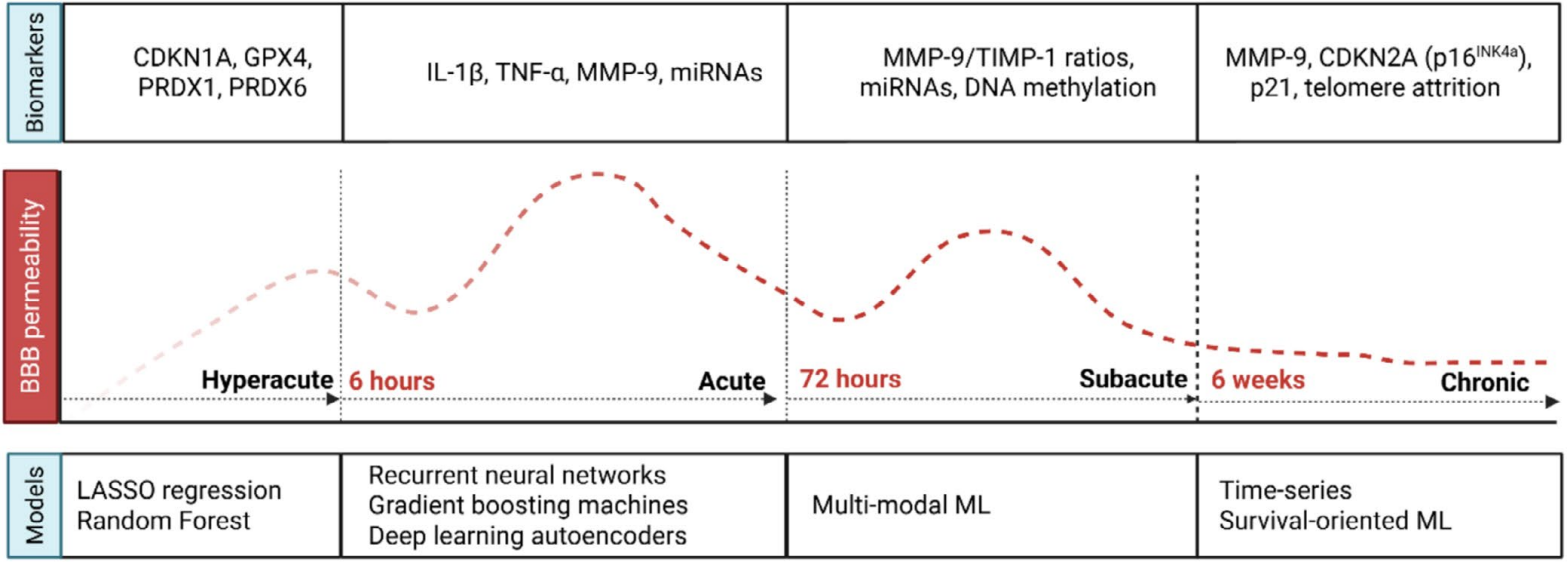
Temporal phases and predictive dynamics of BBB dysfunction after ischemic stroke. BBB permeability follows a biphasic trajectory across ischemic stroke progression, spanning hyperacute, acute, subacute, and chronic phases. The hyperacute phase (within 6 h) is dominated by oxidative stress and cytotoxic injury, reflected by early markers such as CDKN1A, GPX4, PRDX1, and PRDX6. During the acute phase (up to 72 h), inflammatory mediators including IL‐1β, TNF‐α, MMP‐9, and specific miRNAs drive peak BBB disruption. The subacute phase features partial restoration accompanied by remodeling signals—evidenced by MMP‐9/TIMP‐1 ratios, DNA methylation, and persistent miRNA changes. In the chronic phase (beyond 6 weeks), senescence and vascular remodeling become dominant, marked by CDKN2A (p16^INK4a^), p21, and telomere attrition. Corresponding ML models are shown below each phase: LASSO regression and Random Forests are effective for early biomarker identification; Recurrent Neural Networks, Gradient Boosting Machines, and Autoencoders capture dynamic and nonlinear patterns in the acute phase; Multi‐modal ML supports feature fusion in the subacute phase; and Time‐series and Survival‐oriented ML models predict long‐term outcomes and chronic BBB integrity. CDKN1A, cyclin‐dependent kinase inhibitor 1A (p21); GPX4, glutathione peroxidase 4; PRDX1, peroxiredoxin 1; PRDX6, peroxiredoxin 6; IL‐1β, interleukin‐1 beta; TNF‐α, tumor necrosis factor alpha; MMP‐9, matrix metalloproteinase‐9; TIMP‐1, tissue inhibitor of metalloproteinase‐1; miRNA, microRNA; CDKN2A, cyclin‐dependent kinase inhibitor 2A; ML, machine learning; LASSO, least absolute shrinkage and selection operator, blood–brain barrier.

The chronic phase (beyond 6 weeks) emphasizes vascular remodeling, endothelial senescence, and persistent low‐grade neuroinflammation. Markers such as MMP‐9, CDKN2A (p16^INK4a^), p21, and telomere attrition reflect maladaptive repair and vascular stiffness (Figure 3) (Gao et al., 2024; Mathias et al., 2024; Włodarczyk et al., 2024). Time‐series and survival‐oriented machine learning models, such as DeepSurv and Cox‐machine learning, integrate these chronic‐phase signals to predict long‐term outcomes such as recurrent stroke, cognitive decline, and functional recovery (Gao et al., 2023; Li, Kang, et al., 2025; Liang et al., 2025). Thus, these phase‐specific ML approaches enable patient‐specific prognostication, linking mechanistic insight to predictive precision in ischemic stroke management. This temporal modeling approach allows continuous mapping of biological injury trajectories into clinically actionable predictive outputs.

The molecular and cellular features of BBB dysfunction, such as oxidative stress markers, inflammatory cytokines, senescence indicators, and permeability changes, provide measurable inputs for computational modeling. Integrating these mechanistic features with multi‐modal data (imaging, transcriptomics, proteomics, and clinical measures) allows ML algorithms to predict BBB disruption, stroke subtype, and patient‐specific outcomes.

## 3 MULTI‐MODAL DATA INTEGRATION FOR PERSONALIZED PREDICTION

### 3.1 BBB features as multi‐modal ML inputs

Accurate prediction of BBB disruption and ischemic stroke outcomes increasingly depends on integrating heterogeneous data across molecular, cellular, and imaging domains (Sasannia et al., 2024). Key BBB features such as oxidative stress and mitochondrial injury markers (GPX4 and PRDX1/6), inflammatory cytokines (IL‐1β and TNF‐α), senescence indicators (p16 INK4a and telomere attrition), matrix remodeling proteins (MMP‐9), and neuroimaging‐derived metrics (MRI, DCE‐MRI, and ASL) serve as quantifiable indicators of BBB integrity. When combined with multi‐omics profiles (transcriptomics, proteomics, and metabolomics) and clinical data (age, comorbidities, and functional scores), these measures provide a comprehensive representation of BBB status for predictive and mechanistic analyses (Lei et al., 2025; Sasannia et al., 2024). By translating mechanistic BBB insights into quantifiable feature spaces, ML frameworks can capture both static and dynamic dimensions of neurovascular dysfunction. This enables explicit linkage between evolving biomarker patterns, imaging signals, and individualized stroke trajectories, facilitating biologically grounded risk stratification and outcome prediction.

### 3.2 Data integration and feature fusion

Feature fusion is the process of combining different types of data like blood tests and brain scans to get a clearer overall picture than any single source could provide. We can think of it as merging clues from different witnesses to solve a mystery. In practice, there are two main ways to combine this data. You can blend the raw information together from the start (“early fusion”) or you can first analyze each type separately and then combine the results later (“late fusion”). To avoid being overwhelmed by too much data, techniques like principal component analysis (PCA) or autoencoders are used to simplify the information and highlight the most important signals. Finally, methods like SHapley Additive exPlanations (SHAP) or attention mechanisms help make the model's predictions understandable by showing which specific data points, such as a particular biomarker, were most influential in reaching a conclusion (Lovrić et al., 2021). This connects the model's output back to real, interpretable biological or clinical insights (Yi et al., 2023).

Recent studies demonstrate the power of these integrative approaches. Multi‐omics integration using convolutional neural networks (BioCNN) achieved 97.9% accuracy in classifying ischemic stroke subtypes through combined mRNA, miRNA, circRNA, and DNA methylation profiles (Manik, 2023). Stacked multimodal models incorporating clinical and biochemical features successfully predicted stroke risk, while deep learning applied to multi‐channel MRI achieved a Dice Similarity Coefficient of 87.5% for lesion segmentation (Hu et al., 2025; Rahman et al., 2025). Temporal modeling has also advanced, Autoencoder‐LSTM architectures captured dynamic MRI features to forecast stroke outcomes (AUC 0.71 and MAE 0.34), and self‐supervised frameworks integrating 3D brain imaging with clinical data further enhanced personalized risk prediction (Delgrange et al., 2025; Hatami et al., 2023).

**FIGURE 4:**
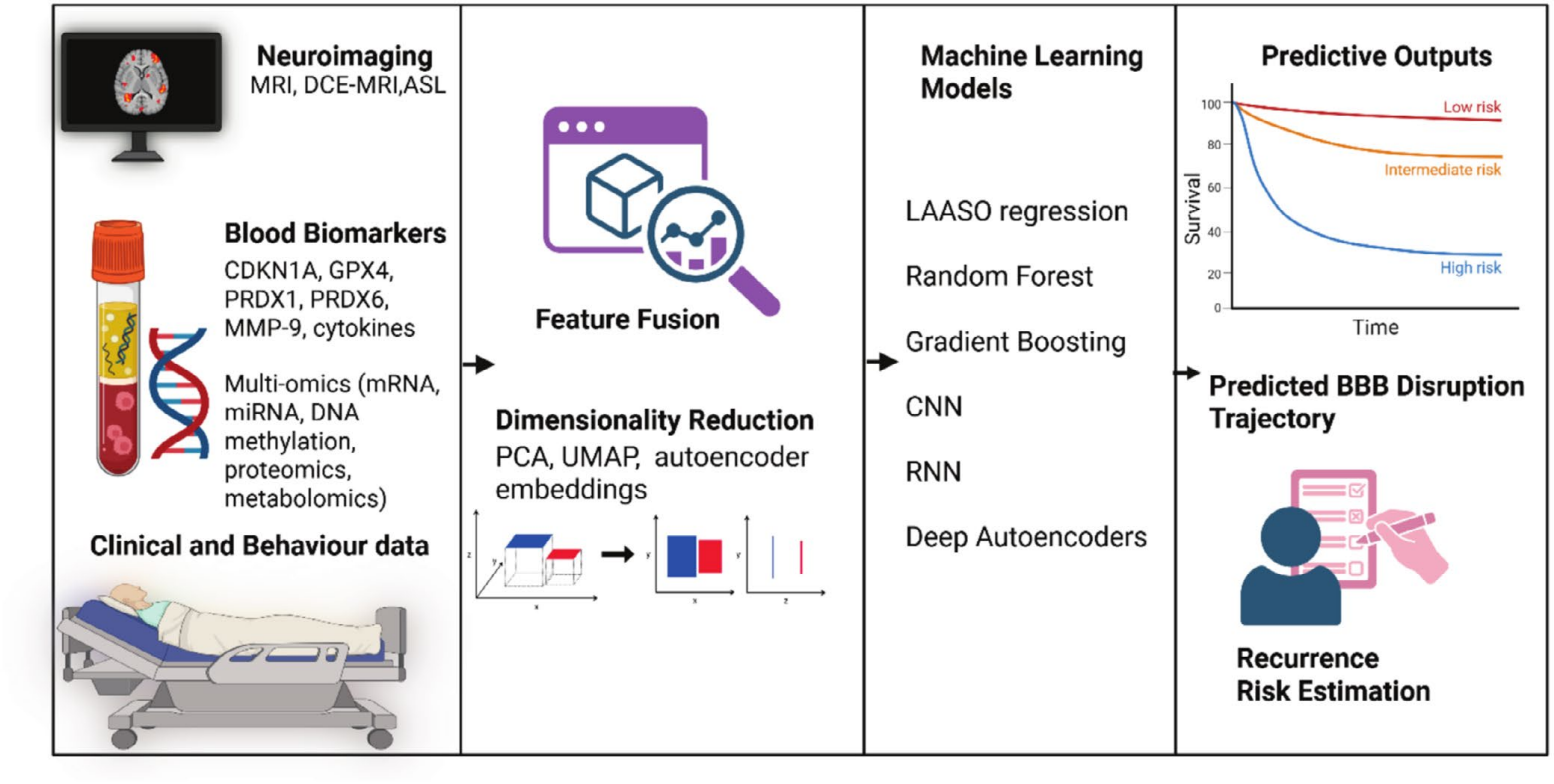
Multi‐modal data integration for personalized prediction of BBB disruption and stroke outcomes. Multi‐modal datasets spanning neuroimaging (MRI, DCE‐MRI, and ASL), circulating biomarkers (CDKN1A, GPX4, PRDX1, PRDX6, MMP‐9, and cytokines), and multi‐omics (mRNA, miRNA, DNA methylation, proteomics, and metabolomics) are fused with clinical and behavioral data through early and late feature fusion strategies. Dimensionality reduction using PCA, UMAP, or autoencoder embeddings enhances signal extraction. Integrated features are modeled by machine learning architectures LASSO regression, Random Forest, Gradient Boosting, CNN, RNN, and deep autoencoders to predict BBB disruption dynamics, stroke subtype, and functional outcomes. Model interpretability (SHAP, attention mechanisms) bridges predictive insights to pathophysiological mechanisms, advancing precision neurovascular medicine. ASL, arterial spin labeling; BBB, blood–brain barrier; CDKN1A, cyclin‐dependent kinase inhibitor 1A; CNN, convolutional neural network; DCE‐MRI, dynamic contrast‐enhanced MRI; GPX4, glutathione peroxidase 4; IL‐1β, interleukin‐1 beta; LASSO, least absolute shrinkage and selection operator; MMP‐9, matrix metalloproteinase‐9; MRI, magnetic resonance imaging; PCA, principal component analysis; PRDX1, peroxiredoxin 1; PRDX6, peroxiredoxin 6; RNN, recurrent neural network; TNF‐α, tumor necrosis factor‐alpha; UMAP, uniform manifold approximation and projection.

Clinical imaging studies have made parallel progress. In stroke lesion segmentation, specially designed networks combine multiple types of MRI and CT perfusion data, achieving high accuracy (up to 85.4%) by improving feature extraction (Gheibi et al., 2023; Rahman et al., 2025). For early stroke identification, models that integrate video and audio recordings (like limb movement and speech) using advanced learning techniques outperform single‐source methods, enabling rapid detection (Ou et al., 2024). Prognosis prediction is improved by ensemble models that merge imaging features with clinical data to better forecast 90‐day outcomes (Jung et al., 2024). Recurrence risk prediction is enhanced by systems that fuse MRI signals with biochemical data using advanced neural networks, achieving up to 95% accuracy, with factors like LDL levels and smoking history as key predictors (Fan et al., 2024). Therefore, multi‐dimensional fusion models, such as those using cross‐modal attention, integrate imaging, clinical data, treatment, and complications to provide cross‐validated, patient‐specific risk assessments (Figure 4).

### 3.3 Mechanistic biomarkers and predictive profiles

Integrating mechanistic biomarkers into these multi‐modal models enables early detection and patient stratification (Zhao et al., 2024). Oxidative stress and mitochondrial injury biomarkers can be combined with neurovascular unit crosstalk markers (IL‐1β, TNF‐α, and exosomal miRNAs) and chronic‐phase vascular remodeling indicators (MMP‐9, p16INK4a, and telomere attrition) to construct comprehensive predictive profiles (Ciaccio & Tuttolomondo, 2023; Huang et al., 2023; Zhang et al., 2024). Machine learning algorithms such as LASSO regression, random forest, gradient boosting, recurrent neural networks, and deep autoencoders can exploit these fused features to model temporal dynamics, predict BBB disruption trajectories, and forecast individualized outcomes such as recurrent stroke or cognitive decline (Zhang et al., 2023). Thus, this integrative approach translates detailed mechanistic insights into actionable, personalized predictions, marking a significant step toward precision neurovascular medicine. Extension of these models to include patient‐specific genetic and epigenetic signatures can improve prediction of BBB vulnerability and therapeutic response. GWAS and multi‐omics studies have identified genetic loci associated with stroke susceptibility and vascular dysfunction (Shehjar et al., 2025). Epigenetic profiling in post‐stroke cerebral microvessels revealed thousands of differentially methylated regions affecting endothelial junction proteins (occludin and claudin‐5), actin remodeling (ezrin), Rho GTPase signaling, and genes driving endothelial‐to‐mesenchymal transition (Sox9 and Snai1), with aging amplifying these effects (Phillips et al., 2023). Integrating these signatures into predictive models enhances precision‐oriented ischemic stroke management by capturing individual BBB repair dynamics.

## 4 MACHINE LEARNING MODEL SELECTION AND VALIDATION

### 4.1 Algorithm selection based on data type

ML model choice is guided by data modality. Tabular biomarkers and clinical variables are commonly analyzed using Random Forest, Gradient Boosting, and LASSO regression, enabling interpretable feature ranking. Imaging datasets are processed using convolutional neural networks, asymmetric U‐Nets, and CNN‐Res architectures for lesion segmentation and BBB permeability estimation. Multimodal integration benefits from attention‐based networks, stacked ensembles, and deep autoencoders that capture cross‐domain interactions while reducing dimensionality.

At the molecular scale, transformer‐based models (MegaMolBART combined with XGBoost) predict BBB permeability from chemical descriptors (SMILES) with an AUC of 0.88, validated in human BBB spheroids. At the patient level, supervised models including Random Forest, Gradient Boosting, and Support Vector Machines integrate clinical and cytokine data to identify biomarkers of BBB disruption, with IL‐6, IL‐5, IL‐10, and IL‐2 ranked as top predictors (Huang, Yang, et al., 2024). Imaging‐based radiomics and perfusion models further associate BBB metrics with hemorrhagic transformation and neurological recovery (Lee, 2024).

### 4.2 Model validation and interpretability

Clinical‐variable‐based stroke prediction studies have leveraged rigorous model comparison and validation frameworks to evaluate algorithms including Logistic Regression, Random Forest, Gradient Boosting, Support Vector Machines, and deep learning networks. For instance, Akinwumi et al. reported that Logistic Regression and Gradient Boosting achieved 95.1% accuracy and a ROC‐AUC of 0.836 using a Kaggle stroke dataset, with Random Forest identifying age, average glucose level, and BMI as top predictors (Akinwumi et al., 2025). Melnykova et al. showed that a Random Forest model trained on balanced data reached 96% accuracy, with precision, recall, and F1‐scores around 90%, underscoring the impact of data preprocessing on predictive performance (Melnykova et al., 2025). Thus, these studies provide a mature foundation for ML‐based BBB research that integrates imaging and clinical data to enhance stroke prognostication (Table 1).

**TABLE 1:** ML models, biomarkers, and predictive performance in BBB and stroke.

ML model/algorithm	Biomarker/data type	Main findings/outcomes	Performance metrics	Advantages	Limitations	References
Transformer‐based Deep Learning (MegaMolBART with XGBoost)	Molecular descriptors, SMILES representations	Predicted BBB permeability	AUC 0.88; validated in human BBB spheroids	Captures molecular‐level interactions; enables high‐throughput prediction	Limited to small‐molecule compounds; not patient‐level	Huang, Yang, et al. (2024)
Random Forest	Clinical and cytokine data (IL‐6, IL‐5, IL‐10, and IL‐2)	Identified key biomarkers of BBB disruption	Not specified	Handles nonlinear relationships; interpretable via feature importance	Requires careful preprocessing; may overfit	Huang, Yang, et al. (2024)
Gradient Boosting	Clinical data	Stroke risk prediction	Accuracy 95.1%; ROC‐AUC 0.836	High predictive accuracy; robust to multicollinearity	Limited external validation	Akinwumi et al. (2025)
Logistic Regression	Clinical data	Stroke risk prediction	Accuracy 95.1%; ROC‐AUC 0.836	Simple, interpretable	Assumes linearity; less flexible with complex interactions	Akinwumi et al. (2025)
Random Forest (balanced dataset)	Clinical data	Stroke risk prediction	Accuracy 96%; Precision, Recall, F1 90%	High performance on imbalanced datasets	Sensitive to hyperparameters; computationally intensive	Melnykova et al. (2025)
BioCNN (Convolutional Neural Network)	Multi‐omics (mRNA, miRNA, circRNA, DNA methylation)	Classified ischemic stroke subtypes	Accuracy 97.9%	Integrates diverse omics data; captures complex interactions	Requires large datasets; limited interpretability	Manik (2023)
Autoencoder‐LSTM	Longitudinal MRI and clinical data	Forecasted stroke outcomes	AUC 0.71; MAE 0.34	Models temporal dynamics	Requires extensive data; complex training	Hatami et al. (2023)
3D CNN integrated with Clinical Data	Multi‐channel MRI	Predicted 90‐day functional outcomes	Accuracy not specified	Integrates imaging and clinical variables	Black‐box nature; needs large dataset	Jung et al. (2024)
Capsule Neural Network and SVM (ISGL)	MRI signals (T1 and T2) and biochemical data	Predicted recurrence risk	Accuracy 95%; key predictors LDL, smoking, and heart disease history	Captures hierarchical features; multimodal integration	Complex architecture; limited external validation	Fan et al. (2024)
Asymmetric U‐Net/CNN‐Res/M2FNet	Multi‐modal MRI and CT perfusion	Stroke lesion segmentation	Dice coefficient up to 85.4%	High segmentation accuracy; robust feature extraction	Computationally demanding; modality‐specific	Gheibi et al. (2023); Rahman et al. (2025)
Multi‐modal Video and Audio (Contrastive Learning)	Limb movement, facial expressions, and speech	Early stroke detection	Not specified	Rapid clinical assessment; multimodal integration	Limited large‐scale validation	Ou et al. (2024)

However, significant gaps remain in translational integration and methodological consistency. Few published models directly combine transcriptomic or proteomic biomarkers of BBB injury with imaging‐based permeability metrics and clinical features within a unified ML pipeline (Nabi et al., 2025). A recent meta‐review highlighted limited external validation, poor calibration reporting, and the underuse of explainable AI (XAI) for mechanistic interpretation (Grant et al., 2025). The absence of multimodal datasets that incorporate molecular, imaging, and clinical layers restricts the capacity of existing models to represent BBB integrity as a dynamic, biologically grounded process during acute ischemic stroke (Li et al., 2024; Nabi et al., 2025). This limitation underscores the need for harmonized pipelines that can capture both the structural and molecular dimensions of BBB pathology while maintaining reproducibility across clinical cohorts.

Emerging best practices from recent comparative and imaging‐radiomics studies recommend a standardized approach to ML model selection and validation (Li et al., 2024; Wang et al., 2025). Future studies are encouraged to evaluate multiple algorithms that include both classical (logistic regression, support vector machines, and random forest) and deep learning models (convolutional neural networks and autoencoders), while performing feature selection within cross‐validation folds to prevent data leakage. Comprehensive model evaluation should include discrimination (AUC), calibration (Brier score), and decision‐curve analysis for clinical utility, complemented by sensitivity and specificity reporting (Grant et al., 2025). Incorporating XAI tools such as SHAP or attention maps facilitates biological interpretability by linking model predictions to measurable mechanisms of BBB disruption (Wang et al., 2025).

Federated learning frameworks additionally enable multi‐center integration of BBB datasets without sharing patient‐level data, enhancing generalizability while preserving privacy (Crowson et al., 2022). Automated hyperparameter optimization coupled with nested cross‐validation further improves model robustness and reproducibility across heterogeneous clinical cohorts (Calle et al., 2025).

## 5 BIOMARKER‐GUIDED THERAPEUTIC DECISION SUPPORT

Integrating multi‐modal biomarker profiles with ML models forms the foundation of precision therapeutics in ischemic stroke (Hu et al., 2025). Recent studies demonstrate that molecular and immune biomarkers can be harnessed through ML to guide individualized therapy. A recent study by Liu et al. (2025) employed integrative ML pipelines to identify blood‐based biomarkers and potential therapeutic agents for ischemic stroke, revealing oxidative, and inflammatory networks predictive of treatment response (Liu et al., 2025). Similarly, Zhao et al. (2024) combined ML with single‐cell transcriptomic analysis to uncover immune and endothelial signatures such as elevated CDKN1A and IL‐1β expression that delineate BBB dysfunction and may inform anti‐inflammatory or redox‐modulating interventions (Zhao et al., 2024). Thus, these findings support the concept that phase‐specific biomarkers of BBB disruption such as oxidative stress mediators, inflammatory cytokines, and senescence indicators (p16^INK4a^, p21, and telomere attrition) can inform therapeutic decision‐making aimed at reducing neuronal injury, mitigating edema, and preserving neurovascular integrity.

ML models further enable dynamic patient stratification based on evolving BBB compromise. In the PRECISE Study, Oliveira et al. (2023) demonstrated that deep learning can extract early‐phase plasma biomarker signatures predictive of infarct expansion and therapeutic responsiveness (Oliveira et al., 2023). Predictive architectures such as recurrent neural networks or survival‐based models (DeepSurv) have shown potential to optimize the timing and intensity of therapeutic interventions by integrating temporal biomarker changes with clinical and imaging data (Klug et al., 2024; Norouzi et al., 2025). Patients exhibiting high ROS and NF‐κB activation may respond to antioxidant or redox‐stabilizing agents, whereas those with pericyte loss or endothelial senescence could benefit from senolytic or vascular‐protective therapies (Li, Ming, et al., 2025; Stojanovic et al., 2025). These ML‐driven approaches complement current stroke management strategies by adapting treatment recommendations to real‐time biological trajectories rather than static clinical variables.

XAI is central to translating biomarker‐guided ML into clinical practice. Interpretable models described by Lee et al. (2023) utilized SHAP and feature attribution maps to visualize how specific biomarkers and imaging variables influence predicted outcomes, thereby improving clinician trust (Lee et al., 2023). Recent evidence emphasizes the integration of interpretability frameworks with treatment‐guidance algorithms to balance predictive power with transparency (Daidone et al., 2023). Visualization of SHAP importance for biomarkers like MMP‐9 or CDKN1A allows mechanistic linkage between predicted benefit and molecular pathways of BBB repair. Therefore, these biomarker‐driven, explainable ML systems provide a path toward precision neurovascular therapy reducing adverse events, optimizing therapeutic timing, and improving long‐term neurological outcomes after ischemic stroke.

## 6 CHALLENGES, LIMITATIONS, AND FUTURE DIRECTIONS

Despite the promise of ML‐driven, biomarker‐informed ischemic stroke management, several challenges limit its immediate clinical translation. Multi‐modal datasets integrating imaging, omics, and clinical parameters are often heterogeneous and incomplete, with substantial variability in imaging protocols, biomarker measurement techniques, and patient demographics. Such heterogeneity complicates model training and hinders generalizability across institutions. Standardization and harmonization of data acquisition and biomarker assays are therefore essential to enhance reproducibility and support multi‐center ML studies (Akinwumi et al., 2025; Daidone et al., 2023).

Another limitation lies in the temporal BBB dysfunction, which evolves throughout the course of ischemic stroke. Accurately modeling these temporal processes requires longitudinal biomarker sampling to capture dynamic molecular and imaging signatures. However, repeated and invasive sampling, particularly cerebrospinal fluid collection, poses significant ethical and logistical barriers, limiting the availability of time‐resolved datasets (Daidone et al., 2023; Liu et al., 2025). Furthermore, small cohort sizes in most omics‐based investigations restrict statistical power and increase the risk of model overfitting. Expanding datasets through multi‐institutional collaboration and federated learning frameworks can mitigate these issues, ensuring model robustness and enhancing predictive validity (Liu et al., 2025).

Interpretability remains a critical barrier to clinical translation. While deep learning models offer superior performance for feature extraction and nonlinear mapping, they often function as “black boxes,” providing limited insight into biological mechanisms or therapeutic decision‐making (Moulaei et al., 2024). XAI techniques such as SHAP values, Grad‐CAM, and attention mechanisms have shown potential to bridge this gap by linking predictions to key mechanistic drivers such as MMP‐9, IL‐6, and CDKN1A, but these approaches require systematic validation in clinical settings to ensure reliability (Daidone et al., 2023; Moulaei et al., 2024).

Looking ahead, several future directions can enhance the translational potential of ML‐driven precision stroke care. First, developing standardized protocols for data collection and biomarker measurement across research centers will improve interoperability and facilitate large‐scale analyses (Akinwumi et al., 2025). Second, integrating multi‐modal and longitudinal datasets that combine clinical, imaging, genetic, and circulating biomarker information will increase model sensitivity and specificity, though this requires advanced data harmonization frameworks (Liu et al., 2025). Third, adopting advanced algorithmic strategies including ensemble learning, transfer learning, and hybrid deep architectures may reduce overfitting and better capture complex neurovascular interactions (Akinwumi et al., 2025). Lastly, expanding patient cohorts and conducting rigorous external validations are essential for ensuring model reliability, clinical credibility, and regulatory acceptance (Daidone et al., 2023) Therefore, addressing these challenges through harmonized data pipelines, transparent and interpretable AI, and collaborative learning consortia will be pivotal in transforming mechanistic insights into actionable, patient‐specific interventions. Such efforts will accelerate the clinical translation of ML‐driven biomarker models toward truly personalized management of ischemic stroke.

## 7 CONCLUSION

The blood–brain barrier is a central regulator of neurovascular homeostasis, and its dysfunction plays a critical role in the pathophysiology of ischemic stroke. Mechanistic insights that include oxidative stress, mitochondrial injury, neurovascular unit crosstalk, vascular remodeling, and endothelial senescence reveal a complex cascade of biological events that can now be characterized through molecular, cellular, and imaging biomarkers. Integrating these multimodal biomarkers with advanced machine learning approaches enables dynamic, patient‐specific prediction of BBB disruption, stroke progression, and long‐term outcomes. Phase‐specific machine learning architectures, such as LASSO regression, Random Forests, recurrent neural networks, and survival‐based frameworks such as DeepSurv, provide robust tools for early risk stratification, trajectory modeling, and personalized therapeutic decision‐making.

Despite challenges related to data heterogeneity, small cohort sizes, and model interpretability, advances in omics platforms, high‐resolution neuroimaging, and explainable artificial intelligence continue to improve reliability and clinical translation. Therefore, machine learning‐guided and biomarker‐informed frameworks represent a transformative step toward precision stroke management by linking mechanistic understanding of blood–brain barrier dysfunction with actionable clinical interventions and personalized neurovascular care.

## AUTHOR CONTRIBUTIONS

**John Nzobokela:** Conceptualization; data curation; formal analysis; methodology; software; validation; visualization. **Lweendo Muchaili:** Data curation; formal analysis; validation; visualization. **Joy Kaluba Mangimela:** Data curation; formal analysis; validation; visualization. **Annet Kirabo:** Data curation; formal analysis; validation; visualization. **Sepiso K. Masenga:** Conceptualization; data curation; formal analysis; funding acquisition; methodology; software; supervision; validation; visualization.

## FUNDING INFORMATION

This work was supported by the Fogarty International Center of the National Institutes of Health, National Institute of Diabetes and Digestive and Kidney Diseases of the National Institutes of Health grants R01HL144941 (AK), 2D43TW009744 (SKM), R21TW012635 (AK and SKM) and the American Heart Association Award Number 24IVPHA1297559 https://doi.org/10.58275/aha.24ivpha1297559.pc.gr.193866 (AK and SKM). The funders had no role in study design, data collection and analysis, decision to publish, or preparation of the manuscript. Its contents are solely the responsibility of the authors and do not necessarily represent official views of the American Heart Association or the National Institutes of Health.

## CONFLICT OF INTEREST STATEMENT

The authors declare that the research was conducted in the absence of any commercial or financial relationships that could be construed as a potential conflict of interest.

## ETHICS STATEMENT

This article is a review of previously published literature and does not involve any new studies with human participants, animals, or identifiable personal data conducted by the authors. Therefore, ethical approval and informed consent were not required for this work. The review was conducted in accordance with principles of academic integrity, with appropriate acknowledgment and citation of all original sources.

## References

[phy271003-bib-0001] Abdullahi, W. , Tripathi, D. , & Ronaldson, P. T. (2018). Blood‐brain barrier dysfunction in ischemic stroke: Targeting tight junctions and transporters for vascular protection. American Journal of Physiology‐Cell Physiology, 315(3), C343–C356. 29949404 10.1152/ajpcell.00095.2018 PMC6171039

[phy271003-bib-0002] Akinwumi, P. O. , Ojo, S. , Nathaniel, T. I. , Wanliss, J. , Karunwi, O. , & Sulaiman, M. (2025). Evaluating machine learning models for stroke prediction based on clinical variables. Frontiers in Neurology, 16, 1668420. 10.3389/fneur.2025.1668420 41018177 PMC12463612

[phy271003-bib-0003] Al‐Jehani, H. M. , Mousa, A. H. , Alhamid, M. A. , & Al‐Mufti, F. (2025). Role of microRNA in the risk stratification of ischemic strokes. Frontiers in Neurology, 16, 1499493. 40012999 10.3389/fneur.2025.1499493 PMC11860075

[phy271003-bib-0004] Angerfors, A. , Brännmark, C. , Lagging, C. , Tai, K. , Månsby Svedberg, R. , Andersson, B. , Jern, C. , & Stanne, T. M. (2023). Proteomic profiling identifies novel inflammation‐related plasma proteins associated with ischemic stroke outcome. Journal of Neuroinflammation, 20(1), 224. 37794467 10.1186/s12974-023-02912-9 PMC10548608

[phy271003-bib-0005] Arbaizar‐Rovirosa, M. , Gallizioli, M. , Lozano, J. J. , Sidorova, J. , Pedragosa, J. , Figuerola, S. , Chaparro‐Cabanillas, N. , Boya, P. , Graupera, M. , Claret, M. , & Urra, X. (2023). Transcriptomics and translatomics identify a robust inflammatory gene signature in brain endothelial cells after ischemic stroke. Journal of Neuroinflammation, 20(1), 207. 37691115 10.1186/s12974-023-02888-6 PMC10494365

[phy271003-bib-0006] Baixauli‐Martín, J. , Burguete, M. C. , López‐Morales, M. A. , Castelló‐Ruiz, M. , Aliena‐Valero, A. , Jover‐Mengual, T. , Falahatgaroshibi, D. , Torregrosa, G. , & Salom, J. B. (2025). Spatio‐temporal characterization of cellular senescence hallmarks in experimental ischemic stroke. International Journal of Molecular Sciences, 26(5), 2364. 40076983 10.3390/ijms26052364 PMC11900039

[phy271003-bib-0007] Balistreri, C. R. , Di Giorgi, L. , & Monastero, R. (2024). Focus of endothelial glycocalyx dysfunction in ischemic stroke and Alzheimer's disease: Possible intervention strategies. Ageing Research Reviews, 99, 102362. 38830545 10.1016/j.arr.2024.102362

[phy271003-bib-0008] Bernardo‐Castro, S. , Sousa, J. A. , Brás, A. , Cecília, C. , Rodrigues, B. , Almendra, L. , Machado, C. , Santo, G. , Silva, F. , Ferreira, L. , & Santana, I. (2020). Pathophysiology of blood–brain barrier permeability throughout the different stages of ischemic stroke and its implication on hemorrhagic transformation and recovery. Frontiers in Neurology, 9(11), 594672. 10.3389/fneur.2020.594672 PMC7756029 33362697

[phy271003-bib-0009] Calle, P. , Bates, A. , Reynolds, J. C. , Liu, Y. , Cui, H. , Ly, S. , Wang, C. , Zhang, Q. , de Armendi, A. J. , Shettarg, S. S. , & Fung, K. M. (2025). Integration of nested cross‐validation, automated hyperparameter optimization, high‐performance computing to reduce and quantify the variance of test performance estimation of deep learning models. Computer Methods and Programs in Biomedicine, 272, 109063. 40946520 10.1016/j.cmpb.2025.109063 PMC12674930

[phy271003-bib-0010] Candelario‐Jalil, E. , Dijkhuizen, R. M. , & Magnus, T. (2022). Neuroinflammation, stroke, blood‐brain barrier dysfunction, and imaging modalities. Stroke, 53(5), 1473–1486. 35387495 10.1161/STROKEAHA.122.036946 PMC9038693

[phy271003-bib-0011] Chen, X. , Threlkeld, S. W. , Cummings, E. E. , Juan, I. , Makeyev, O. , Besio, W. G. , Gaitanis, J. , Banks, W. A. , Sadowska, G. B. , & Stonestreet, B. S. (2012). Ischemia‐reperfusion impairs blood‐brain barrier function and alters tight junction protein expression in the ovine fetus. Neuroscience, 226, 89–100. 22986172 10.1016/j.neuroscience.2012.08.043 PMC3490041

[phy271003-bib-0012] Ciaccio, A. M. , & Tuttolomondo, A. (2023). Exosomal miRNAs as biomarkers of ischemic stroke. Brain Sciences, 13(12), 1647. 38137095 10.3390/brainsci13121647 PMC10741776

[phy271003-bib-0013] Crowson, M. G. , Moukheiber, D. , Arévalo, A. R. , Lam, B. D. , Mantena, S. , Rana, A. , Goss, D. , Bates, D. W. , & Celi, L. A. (2022). A systematic review of federated learning applications for biomedical data. PLoS Digital Health, 1(5), e0000033. 36812504 10.1371/journal.pdig.0000033 PMC9931322

[phy271003-bib-0014] Ćurko‐Cofek, B. , Jenko, M. , Taleska Stupica, G. , Batičić, L. , Krsek, A. , Batinac, T. , Ljubačev, A. , Zdravković, M. , Knežević, D. , Šoštarič, M. , & Sotošek, V. (2024). The crucial triad: Endothelial glycocalyx, oxidative stress, and inflammation in cardiac surgery—Exploring the molecular connections. International Journal of Molecular Sciences, 25(20), 10891. 39456673 10.3390/ijms252010891 PMC11508174

[phy271003-bib-0015] Daidone, M. , Ferrantelli, S. , & Tuttolomondo, A. (2023). Machine learning applications in stroke medicine: Advancements, challenges, and future prospectives. Neural Regeneration Research, 19(4), 769. 10.4103/1673-5374.382228 PMC10664112 37843210

[phy271003-bib-0016] Dancy, C. , Heintzelman, K. E. , & Katt, M. E. (2024). The glycocalyx: The importance of sugar coating the blood‐brain barrier. International Journal of Molecular Sciences, 25(15), 8404. 39125975 10.3390/ijms25158404 PMC11312458

[phy271003-bib-0017] Delgrange, C. , Demler, O. , Mora, S. , Menze, B. , de la Rosa, E. , & Davoudi, N. (2025). Advancing stroke risk prediction using a multi‐modal foundation model. arXiv. http://arxiv.org/abs/2411.09822

[phy271003-bib-0018] Emmerich, J. , Chanpura, A. , Lu, T. , Baird, A. , Barone, F. , Gustafson, D. , Siepmann, T. , Huttner, H. , & Barlinn, K. (2025). Blood‐based MMP‐9 for the early differentiation of acute ischemic stroke: A systematic review and meta‐analysis. Journal of Stroke and Cerebrovascular Diseases, 34(11), 108454. 40992445 10.1016/j.jstrokecerebrovasdis.2025.108454

[phy271003-bib-0019] Fan, D. , Miao, R. , Huang, H. , Wang, X. , Li, S. , Huang, Q. , Yang, S. , & Deng, R. (2024). Multimodal ischemic stroke recurrence prediction model based on the capsule neural network and support vector machine. Medicine (Baltimore), 103(35), e39217. 39213233 10.1097/MD.0000000000039217 PMC11365640

[phy271003-bib-0020] Feigin, V. L. , Brainin, M. , Norrving, B. , Martins, S. O. , Pandian, J. , Lindsay, P. , F Grupper, M. , & Rautalin, I. (2025). World stroke organization: Global stroke fact sheet 2025. International Journal of Stroke, 20(2), 132–144. 39635884 10.1177/17474930241308142 PMC11786524

[phy271003-bib-0021] Feigin, V. L. , Owolabi, M. O. , & World Stroke Organization–Lancet Neurology Commission Stroke Collaboration Group . (2023). Pragmatic solutions to reduce the global burden of stroke: A world stroke organization‐lancet neurology commission. Lancet Neurology, 22(12), 1160–1206. 37827183 10.1016/S1474-4422(23)00277-6 PMC10715732

[phy271003-bib-0022] Fong, H. , Zhou, B. , Feng, H. , Luo, C. , Bai, B. , Zhang, J. , & Wang, Y. (2024). Recapitulation of structure–function–regulation of blood–brain barrier under (Patho)physiological conditions. Cells, 13(3), 260. 38334652 10.3390/cells13030260 PMC10854731

[phy271003-bib-0023] French, S. R. , Meyer, B. P. , Arias, J. C. , Levendovzsky, S. R. , & Weinkauf, C. C. (2025). Biomarkers of blood–brain barrier and neurovascular unit integrity in human cognitive impairment and dementia. Alzheimer's & Dementia, 21(3), e70104. 10.1002/alz.70104 PMC11947770 40145342

[phy271003-bib-0024] Gao, H. M. , Chen, H. , Cui, G. Y. , & Hu, J. X. (2023). Damage mechanism and therapy progress of the blood‐brain barrier after ischemic stroke. Cell & Bioscience, 13(1), 1–13. 37915036 10.1186/s13578-023-01126-z PMC10619327

[phy271003-bib-0025] Gao, Z. , Santos, R. B. , Rupert, J. , Van Drunen, R. , Yu, Y. , Eckel‐Mahan, K. , & Kolonin, M. G. (2024). Endothelial‐specific telomerase inactivation causes telomere‐independent cell senescence and multi‐organ dysfunction characteristic of aging. Aging Cell, 23(6), e14138. 38475941 10.1111/acel.14138 PMC11296101

[phy271003-bib-0026] Geranmayeh, M. H. , Rahbarghazi, R. , & Farhoudi, M. (2019). Targeting pericytes for neurovascular regeneration. Cell Communication and Signaling: CCS, 17(1), 26. 30894190 10.1186/s12964-019-0340-8 PMC6425710

[phy271003-bib-0027] Gheibi, Y. , Shirini, K. , Razavi, S. N. , Farhoudi, M. , & Samad‐Soltani, T. (2023). CNN‐res: Deep learning framework for segmentation of acute ischemic stroke lesions on multimodal MRI images. BMC Medical Informatics and Decision Making, 23(1), 1–14. 37752508 10.1186/s12911-023-02289-y PMC10521570

[phy271003-bib-0028] Grant, N. , Machado Reyes, D. , Yang, Z. , Wan, L. , Wang, C. , & Yan, P. (2025). Blood brain barrier permeability prediction with artificial intelligence and machine learning: A meta‐review and future directions. Discover Artificial Intelligence, 5(1), 254.

[phy271003-bib-0029] Gülke, E. , Gelderblom, M. , & Magnus, T. (2018). Danger signals in stroke and their role on microglia activation after ischemia. Therapeutic Advances in Neurological Disorders, 11, 1756286418774254. 29854002 10.1177/1756286418774254 PMC5968660

[phy271003-bib-0030] Guo, X. , Liu, R. , Jia, M. , Wang, Q. , & Wu, J. (2023). Ischemia reperfusion injury induced blood brain barrier dysfunction and the involved molecular mechanism. Neurochemical Research, 48(8), 2320–2334. 37017889 10.1007/s11064-023-03923-x

[phy271003-bib-0031] Han, Y. , & Kim, S. Y. (2023). Endothelial senescence in vascular diseases: Current understanding and future opportunities in senotherapeutics. Experimental & Molecular Medicine, 55(1), 1–12. 36599934 10.1038/s12276-022-00906-w PMC9898542

[phy271003-bib-0032] Hansen, L. M. B. , Dam, V. S. , Guldbrandsen, H. Ø. , Staehr, C. , Pedersen, T. M. , Kalucka, J. M. , Beck, H. C. , Postnov, D. D. , Lin, L. , & Matchkov, V. V. (2025). Spatial transcriptomics and proteomics profiling after ischemic stroke reperfusion: Insights into vascular alterations. Stroke, 56(4), 1036–1047. 40052263 10.1161/STROKEAHA.124.048085

[phy271003-bib-0033] Hatami, N. , Mechtouff, L. , Rousseau, D. , Cho, T. H. , Eker, O. , Berthezene, Y. , & Frindel, C. (2023). A novel autoencoders‐LSTM model for stroke outcome prediction using multimodal MRI data. arXiv. http://arxiv.org/abs/2303.09484

[phy271003-bib-0034] Hosoya, K. , Ohtsuki, S. , & Terasaki, T. (2002). Recent advances in the brain‐to‐blood efflux transport across the blood‐brain barrier. International Journal of Pharmaceutics, 248(1–2), 15–29. 12429456 10.1016/s0378-5173(02)00457-x

[phy271003-bib-0035] Hu, B. , Chen, X. , Chen, T. , Xu, T. , Cao, Y. , Sun, J. , Chen, X. , Chen, S. , & Chen, K. (2025). Multimodal machine learning‐based marker enables the link between obesity‐related indices and future stroke: A prospective cohort study. EClinicalMedicine, 85, 103331. 40678699 10.1016/j.eclinm.2025.103331 PMC12269863

[phy271003-bib-0036] Hu, X. , Bao, Y. , Li, M. , Zhang, W. , & Chen, C. (2024). The role of ferroptosis and its mechanism in ischemic stroke. Experimental Neurology, 372, 114630. 38056585 10.1016/j.expneurol.2023.114630

[phy271003-bib-0037] Huang, E. T. C. , Yang, J. S. , Liao, K. Y. K. , Tseng, W. C. W. , Lee, C. K. , Gill, M. , Compas, C. , See, S. , & Tsai, F. J. (2024). Predicting blood–brain barrier permeability of molecules with a large language model and machine learning. Scientific Reports, 14(1), 15844. 38982309 10.1038/s41598-024-66897-y PMC11233737

[phy271003-bib-0038] Huang, Y. , Wang, Z. , Huang, Z. X. , & Liu, Z. (2023). Biomarkers and the outcomes of ischemic stroke. Frontiers in Molecular Neuroscience, 16, 1171101. 10.3389/fnmol.2023.1171101 37342100 PMC10277488

[phy271003-bib-0039] Huang, Z. , Xu, P. , Hess, D. C. , & Zhang, Q. (2024). Cellular senescence as a key contributor to secondary neurodegeneration in traumatic brain injury and stroke. Translational Neurodegeneration, 12(13), 61. 10.1186/s40035-024-00457-2 PMC11636056 39668354

[phy271003-bib-0040] Jafarpour, S. , Asefzadeh, M. , & Aboutaleb, E. (2024). The application of machine learning in predicting the permeability of drugs across the blood brain barrier. Iranian Journal of Pharmaceutical Research: IJPR, 23(1), e149367. 40066117 10.5812/ijpr-149367 PMC11892787

[phy271003-bib-0041] Jin, J. , Fang, F. , Gao, W. , Chen, H. , Wen, J. , Wen, X. , & Chen, J. (2021). The structure and function of the glycocalyx and its connection with blood‐brain barrier. Frontiers in Cellular Neuroscience, 15, 739699. 10.3389/fncel.2021.739699 34690703 PMC8529036

[phy271003-bib-0042] Jung, H. S. , Lee, E. J. , Chang, D. I. , Cho, H. J. , Lee, J. , Cha, J. K. , Park, M. S. , Yu, K. H. , Jung, J. M. , Ahn, S. H. , Kim, D. E. , Lee, J. H. , Hong, K. S. , Sohn, S. I. , Park, K. P. , Kwon, S. U. , Kim, J. S. , Chang, J. Y. , Kim, B. J. , … KOSNI Investigators . (2024). A multimodal ensemble deep learning model for functional outcome prognosis of stroke patients. Journal of Stroke, 26(2), 312–320. 38836278 10.5853/jos.2023.03426 PMC11164594

[phy271003-bib-0043] Kadry, H. , Noorani, B. , & Cucullo, L. (2020). A blood–brain barrier overview on structure, function, impairment, and biomarkers of integrity. Fluids and Barriers of the CNS, 17(1), 69. 33208141 10.1186/s12987-020-00230-3 PMC7672931

[phy271003-bib-0044] Klug, J. , Leclerc, G. , Dirren, E. , & Carrera, E. (2024). Machine learning for early dynamic prediction of functional outcome after stroke. Communication & Medicine, 4(1), 232. 10.1038/s43856-024-00666-w PMC11561255 39537988

[phy271003-bib-0045] Knox, E. G. , Aburto, M. R. , Clarke, G. , Cryan, J. F. , & O'Driscoll, C. M. (2022). The blood‐brain barrier in aging and neurodegeneration. Molecular Psychiatry, 27(6), 2659–2673. 35361905 10.1038/s41380-022-01511-z PMC9156404

[phy271003-bib-0046] Ko, K. , Suzuki, T. , Ishikawa, R. , Hattori, N. , Ito, R. , Umehara, K. , Furihata, T. , Dohmae, N. , Linhardt, R. J. , Igarashi, K. , & Toida, T. (2021). Ischemic stroke disrupts the endothelial glycocalyx through activation of proHPSE via acrolein exposure. The Journal of Biological Chemistry, 295(52), 18614–18624. 10.1074/jbc.RA120.015105 PMC7939480 33127645

[phy271003-bib-0047] Kousar, T. , Rahim, M. S. M. , Iqbal, S. , Yousaf, F. , & Sanaullah, M. (2025). Applications of deep learning algorithms in ischemic stroke detection, segmentation, and classification. Artificial Intelligence Review, 58(5), 149.

[phy271003-bib-0048] Lee, J. , Park, K. M. , & Park, S. (2023). Interpretable machine learning for prediction of clinical outcomes in acute ischemic stroke. Frontiers in Neurology, 14, 1234046. 37745661 10.3389/fneur.2023.1234046 PMC10513028

[phy271003-bib-0049] Lee, T. Y. (2024). Deep learning to predict functional outcome in acute ischemic stroke. Radiology, 313(1), e242705. 39404635 10.1148/radiol.242705

[phy271003-bib-0050] Lei, W. , Zhuang, H. , Huang, W. , & Sun, J. (2025). Neuroinflammation and energy metabolism: A dual perspective on ischemic stroke. Journal of Translational Medicine, 23(1), 413. 40211331 10.1186/s12967-025-06440-3 PMC11983748

[phy271003-bib-0051] Li, B. , Ming, H. , Qin, S. , Nice, E. C. , Dong, J. , Du, Z. , & Huang, C. (2025). Redox regulation: Mechanisms, biology and therapeutic targets in diseases. Signal Transduction and Targeted Therapy, 10(1), 72. 40050273 10.1038/s41392-024-02095-6 PMC11885647

[phy271003-bib-0052] Li, G. , Zhao, Y. , Ma, W. , Gao, Y. , & Zhao, C. (2024). Systems‐level computational modeling in ischemic stroke: From cells to patients. Frontiers in Physiology, 15, 1394740. 39015225 10.3389/fphys.2024.1394740 PMC11250596

[phy271003-bib-0053] Li, S. S. , Wu, J. J. , Xing, X. X. , Li, Y. L. , Ma, J. , Duan, Y. J. , Zhang, J. P. , Shan, C. L. , Hua, X. Y. , Zheng, M. X. , & Xu, J. G. (2023). Focal ischemic stroke modifies microglia‐derived exosomal miRNAs: Potential role of mir‐212‐5p in neuronal protection and functional recovery. Biological Research, 56(1), 52. 37789455 10.1186/s40659-023-00458-x PMC10548705

[phy271003-bib-0054] Li, T. , Kang, X. , Zhang, S. , Wang, Y. , He, J. , Li, H. , Shao, C. , & Kang, J. (2025). Integrating machine learning and multi‐omics analysis to reveal nucleotide metabolism‐related immune genes and their functional validation in ischemic stroke. Frontiers in Immunology, 16, 1561544. 40207230 10.3389/fimmu.2025.1561544 PMC11979214

[phy271003-bib-0055] Liang, Y. , Chen, J. , Chen, Y. , Tong, Y. , Li, L. , Xu, Y. , & Wu, S. (2025). Advances in the detection of biomarkers for ischemic stroke. Frontiers in Neurology, 24(16), 1488726. 10.3389/fneur.2025.1488726 PMC11891058 40066310

[phy271003-bib-0056] Liu, J. , Bai, C. , Yang, H. , Song, L. , Xu, H. , Sun, Y. , Suo, M. , Gao, Z. , Li, H. , Wang, F. , & Chen, J. (2025). Machine learning‐driven identification of blood‐based biomarkers and therapeutic agents for personalized ischemic stroke management. Journal of Cardiovascular Translational Research, 18(4), 924–940. 40694178 10.1007/s12265-025-10635-w PMC12436529

[phy271003-bib-0057] Liu, L. , Zhang, L. , Feng, H. , Li, S. , Liu, M. , Zhao, J. , & Liu, H. (2021). Prediction of the blood‐brain barrier (BBB) permeability of chemicals based on machine‐learning and ensemble methods. Chemical Research in Toxicology, 34(6), 1456–1467. 34047182 10.1021/acs.chemrestox.0c00343

[phy271003-bib-0058] Lovrić, M. , Đuričić, T. , Tran, H. T. N. , Hussain, H. , Lacić, E. , Rasmussen, M. A. , & Kern, R. (2021). Should we embed in chemistry? A comparison of unsupervised transfer learning with PCA, UMAP, and VAE on molecular fingerprints. Pharmaceuticals, 14(8), 758. 34451855 10.3390/ph14080758 PMC8400160

[phy271003-bib-0059] Manik, M. M. T. G. (2023). Multi‐omics integration with machine learning for early detection of ischemic stroke through biomarkers discovery. Journal of Ecohumanism, 2(2), 175–187.

[phy271003-bib-0060] Masenga, S. K. , & Kirabo, A. (2024). The NLRP3 inflammasome in ischemic stroke. Frontiers in Stroke, 3, 1382379. 10.3389/fstro.2024.1382379 41542272 PMC12802677

[phy271003-bib-0061] Mathias, K. , Machado, R. S. , Stork, S. , dos Santos, D. , Joaquim, L. , Generoso, J. , Danielski, L. G. , Barichello, T. , Prophiro, J. S. , & Petronilho, F. (2024). Blood‐brain barrier permeability in the ischemic stroke: An update. Microvascular Research, 151, 104621. 37918521 10.1016/j.mvr.2023.104621

[phy271003-bib-0062] Mayer, M. G. , & Fischer, T. (2024). Microglia at the blood brain barrier in health and disease. Frontiers in Cellular Neuroscience, 18, 1360195. 38550920 10.3389/fncel.2024.1360195 PMC10976855

[phy271003-bib-0063] Melnykova, N. , Patereha, Y. , Skopivskyi, S. , Farion, M. , Fedushko, S. , & Drohomyretska, K. (2025). Machine learning for stroke prediction using imbalanced data. Scientific Reports, 15(1), 33773. 41027935 10.1038/s41598-025-01855-w PMC12484691

[phy271003-bib-0064] Moulaei, K. , Afshari, L. , Moulaei, R. , Sabet, B. , Mousavi, S. M. , & Afrash, M. R. (2024). Explainable artificial intelligence for stroke prediction through comparison of deep learning and machine learning models. Scientific Reports, 14(1), 31392. 39733046 10.1038/s41598-024-82931-5 PMC11682355

[phy271003-bib-0065] Müller, S. , Kufner, A. , Dell'Orco, A. , Rackoll, T. , Mekle, R. , Piper, S. K. , Fiebach, J. B. , Villringer, K. , Flöel, A. , Endres, M. , Ebinger, M. , & Nave, A. H. (2021). Evolution of blood‐brain barrier permeability in subacute ischemic stroke and associations with serum biomarkers and functional outcome. Frontiers in Neurology, 12, 730923. 34744972 10.3389/fneur.2021.730923 PMC8567961

[phy271003-bib-0066] Nabi, A. E. , Pouladvand, P. , Liu, L. , Hua, N. , & Ayubcha, C. (2025). Machine learning in drug development for neurological diseases: A review of blood brain barrier permeability prediction models. Molecular Informatics, 44(3), e202400325. 40146590 10.1002/minf.202400325 PMC11949286

[phy271003-bib-0067] Nian, K. , Harding, I. C. , Herman, I. M. , & Ebong, E. E. (2020). Blood‐brain barrier damage in ischemic stroke and its regulation by endothelial Mechanotransduction. Frontiers in Physiology, 11, 605398. 10.3389/fphys.2020.605398 33424628 PMC7793645

[phy271003-bib-0068] Norouzi, S. , Ahmadi, S. , Alinia, S. , Farzipoor, F. , Shahsavari, A. , Hajizadeh, E. , & Jafarabadi, M. A. (2025). Machine learning predictive models for survival in patients with brain stroke. Health Promotion Perspective, 15(1), 63–72. 10.34172/hpp.025.43635 PMC12125501 40453683

[phy271003-bib-0069] O'Hare, N. , Millican, K. , & Ebong, E. E. (2024). Unraveling neurovascular mysteries: The role of endothelial glycocalyx dysfunction in Alzheimer's disease pathogenesis. Frontiers in Physiology, 15, 1394725. 10.3389/fphys.2024.1394725 39027900 PMC11254711

[phy271003-bib-0070] Oliveira, G. , Fonseca, A. C. , Ferro, J. , & Oliveira, A. L. (2023). Deep learning‐based extraction of biomarkers for the prediction of the functional outcome of ischemic stroke patients. Diagnostics, 13(24), 3604. 38132189 10.3390/diagnostics13243604 PMC10743068

[phy271003-bib-0071] Ou, Z. , Wang, H. , Zhang, B. , Liang, H. , Hu, B. , Ren, L. , Liu, Y. , Zhang, Y. , Dai, C. , Wu, H. , & Li, W. (2024). Early identification of stroke through deep learning with multi‐modal human speech and movement data. Neural Regeneration Research, 20(1), 234–241. 38767488 10.4103/1673-5374.393103 PMC11246124

[phy271003-bib-0072] Pacinella, G. , Bona, M. M. , Todaro, F. , Ciaccio, A. M. , Daidone, M. , & Tuttolomondo, A. (2025). Tracing inflammation in ischemic stroke: Biomarkers and clinical insight. International Journal of Molecular Sciences, 26(19), 9801. 41097065 10.3390/ijms26199801 PMC12525443

[phy271003-bib-0073] Pawluk, H. , Tafelska‐Kaczmarek, A. , Sopońska, M. , Porzych, M. , Modrzejewska, M. , Pawluk, M. , Kurhaluk, N. , Tkaczenko, H. , & Kołodziejska, R. (2024). The influence of oxidative stress markers in patients with ischemic stroke. Biomolecules, 14(9), 1130. 39334896 10.3390/biom14091130 PMC11430825

[phy271003-bib-0074] Phillips, C. M. , Stamatovic, S. M. , Keep, R. F. , & Andjelkovic, A. V. (2023). Epigenetics and stroke: Role of DNA methylation and effect of aging on blood‐brain barrier recovery. Fluids Barriers CNS, 20(1), 14. 10.1186/s12987-023-00414-7 36855111 PMC9972738

[phy271003-bib-0075] Picos, A. , Seoane, N. , Campos‐Toimil, M. , & Viña, D. (2025). Vascular senescence and aging: Mechanisms, clinical implications, and therapeutic prospects. Biogerontology, 26(3), 118. 40418230 10.1007/s10522-025-10256-5 PMC12106568

[phy271003-bib-0076] Protzmann, J. , Jung, F. , Jakobsson, L. , & Fredriksson, L. (2024). Analysis of ischemic stroke‐mediated effects on blood–brain barrier properties along the arteriovenous axis assessed by intravital two‐photon imaging. Fluids and Barriers of the CNS, 15(21), 35. 10.1186/s12987-024-00537-5 PMC11017501 38622710

[phy271003-bib-0077] Rahman, A. , Chowdhury, M. E. H. , Wadud, M. S. I. , Sarmun, R. , Mushtak, A. , Zoghoul, S. B. , & Al‐Hashimi, I. (2025). Deep learning‐driven segmentation of ischemic stroke lesions using Multi‐Channel MRI. arXiv. http://arxiv.org/abs/2501.02287

[phy271003-bib-0078] Rahmig, J. , Chanpura, A. , Schultz, A. , Barone, F. C. , Gustafson, D. , & Baird, A. E. (2024). Blood‐based protein biomarkers during the acute ischemic stroke treatment window: A systematic review. Frontiers in Neurology, 15, 1411307. 10.3389/fneur.2024.1411307 39091977 PMC11291248

[phy271003-bib-0079] Real, M. G. C. , Falcione, S. R. , Boghozian, R. , Clarke, M. , Todoran, R. , St Pierre, A. , Zhang, Y. , Joy, T. , & Jickling, G. C. (2024). Endothelial cell senescence effect on the blood‐brain barrier in stroke and cognitive impairment. Neurology, 103(11), e210063. 39541552 10.1212/WNL.0000000000210063

[phy271003-bib-0080] Sasannia, S. , Leigh, R. , Bastani, P. B. , Shin, H. G. , van Zijl, P. , Knutsson, L. , & Nyquist, P. (2024). Blood‐brain barrier breakdown in brain ischemia: Insights from MRI perfusion imaging. Neurotherapeutics, 22(1), e00516. 39709246 10.1016/j.neurot.2024.e00516 PMC11840350

[phy271003-bib-0081] Shehjar, F. , Mahajan, R. , Shahnaz, S. , & Shah, Z. A. (2025). Genetic and epigenetic architectures of stroke: Insights from GWAS to precision medicine. Neurochemistry International, 190, 106059. 41005670 10.1016/j.neuint.2025.106059 PMC13261748

[phy271003-bib-0082] Shi, S. M. , Suh, R. J. , Shon, D. J. , Garcia, F. J. , Buff, J. K. , Atkins, M. , Li, L. , Lu, N. , Sun, B. , Luo, J. , To, N. S. , Cheung, T. H. , McNerney, M. W. , Heiman, M. , Bertozzi, C. R. , & Wyss‐Coray, T. (2025). Glycocalyx dysregulation impairs blood‐brain barrier in ageing and disease. Nature, 639(8056), 985–994. 40011765 10.1038/s41586-025-08589-9 PMC11946907

[phy271003-bib-0083] Stojanovic, B. , Jovanovic, I. , Dimitrijevic Stojanovic, M. , Stojanovic, B. S. , Kovacevic, V. , Radosavljevic, I. , Jovanovic, D. , Miletic Kovacevic, M. , Zornic, N. , Arsic, A. A. , Eric, S. , Mirkovic, N. , Nesic, J. , Jakovljevic, S. , Lazarevic, S. , Milivojcevic Bevc, I. , & Milosevic, B. (2025). Oxidative stress‐driven cellular senescence: Mechanistic crosstalk and therapeutic horizons. Antioxidants, 14(8), 987. 40867884 10.3390/antiox14080987 PMC12383077

[phy271003-bib-0084] Tian, X. , Li, X. , Pan, M. , Yang, L. Z. , Li, Y. , & Fang, W. (2024). Progress of ferroptosis in ischemic stroke and therapeutic targets. Cellular and Molecular Neurobiology, 44, 25. 38393376 10.1007/s10571-024-01457-6 PMC10891262

[phy271003-bib-0085] Turner, R. J. , & Sharp, F. R. (2016). Implications of MMP9 for blood brain barrier disruption and hemorrhagic transformation following ischemic stroke. Frontiers in Cellular Neuroscience, 10, 56. 10.3389/fncel.2016.00056 26973468 PMC4777722

[phy271003-bib-0086] Venkat, P. , Chopp, M. , & Chen, J. (2017). Blood–brain barrier disruption, vascular impairment, and ischemia/reperfusion damage in diabetic stroke. Journal of the American Heart Association: Cardiovascular and Cerebrovascular Disease, 6(6), e005819. 10.1161/JAHA.117.005819 PMC5669184 28572280

[phy271003-bib-0087] Wang, L. , Zhang, X. , Xiong, X. , Zhu, H. , Chen, R. , Zhang, S. , Chen, G. , & Jian, Z. (2022). Nrf2 regulates oxidative stress and its role in cerebral ischemic stroke. Antioxidants, 11(12), 2377. 36552584 10.3390/antiox11122377 PMC9774301

[phy271003-bib-0088] Wang, Z. , Jiang, C. , Zhang, X. , Mu, T. , Li, Q. , Wang, S. , Dong, C. , Shen, Y. , Dai, Z. , & Chen, F. (2025). Machine learning‐based prognostic prediction for acute ischemic stroke using whole‐brain and infarct multi‐PLD ASL radiomics. BMC Medical Imaging, 25(1), 267. 40615945 10.1186/s12880-025-01807-w PMC12228200

[phy271003-bib-0089] Włodarczyk, L. , Cichon, N. , Karbownik, M. S. , Saluk, J. , & Miller, E. (2024). Exploring the role of MMP‐9 and MMP‐9/TIMP‐1 ratio in subacute stroke recovery: A prospective observational study. International Journal of Molecular Sciences, 25(11), 5745. 38891934 10.3390/ijms25115745 PMC11172289

[phy271003-bib-0090] Yetim, E. , Topcuoglu, M. A. , Yurur Kutlay, N. , Tukun, A. , Oguz, K. K. , & Arsava, E. M. (2021). The association between telomere length and ischemic stroke risk and phenotype. Scientific Reports, 26(11), 10967. 10.1038/s41598-021-90435-9 PMC8155040 34040069

[phy271003-bib-0091] Yi, F. , Yang, H. , Chen, D. , Qin, Y. , Han, H. , Cui, J. , Bai, W. , Ma, Y. , Zhang, R. , & Yu, H. (2023). XGBoost‐SHAP‐based interpretable diagnostic framework for alzheimer's disease. BMC Medical Informatics and Decision Making, 23(1), 137. 37491248 10.1186/s12911-023-02238-9 PMC10369804

[phy271003-bib-0092] Zhang, L. M. , Liang, X. L. , Xiong, G. F. , Xing, X. L. , Zhang, Q. J. , Zhang, B. R. , & Liu, M. W. (2024). Analysis and identification of oxidative stress‐ferroptosis related biomarkers in ischemic stroke. Scientific Reports, 14(1), 3803. 38360841 10.1038/s41598-024-54555-2 PMC10869843

[phy271003-bib-0093] Zhang, X. , Wang, X. , Wang, S. , Zhang, Y. , Wang, Z. , Yang, Q. , Wang, S. , Cao, R. , Yu, B. , Zheng, Y. , & Dang, Y. (2023). Machine learning algorithms assisted identification of post‐stroke depression associated biological features. Frontiers in Neuroscience, 17, 1146620. 10.3389/fnins.2023.1146620 36968495 PMC10030717

[phy271003-bib-0094] Zhao, Y. , Ma, X. , Meng, X. , Li, H. , & Tang, Q. (2024). Integrating machine learning and single‐cell transcriptomic analysis to identify potential biomarkers and analyze immune features of ischemic stroke. Scientific Reports, 14(1), 26069. 39478056 10.1038/s41598-024-77495-3 PMC11525974

[phy271003-bib-0095] Zhi, S. , Hu, X. , Ding, Y. , Chen, H. , Li, X. , Tao, Y. , & Li, W. (2024). An exploration on the machine‐learning‐based stroke prediction model. Frontiers in Neurology, 15, 1372431. 10.3389/fneur.2024.1372431 38742047 PMC11089140

[phy271003-bib-0096] Zhu, J. , Li, X. , Yin, J. , Hu, Y. , Gu, Y. , & Pan, S. (2018). Glycocalyx degradation leads to blood–brain barrier dysfunction and brain edema after asphyxia cardiac arrest in rats. Journal of Cerebral Blood Flow and Metabolism, 38(11), 1979–1992. 28825336 10.1177/0271678X17726062 PMC6259325

[phy271003-bib-0097] Zhu, J. , Li, Z. , Ji, Z. , Wu, Y. , He, Y. , Liu, K. , Chang, Y. , Peng, Y. , Lin, Z. , Wang, S. , & Wang, D. (2022). Glycocalyx is critical for blood‐brain barrier integrity by suppressing caveolin1‐dependent endothelial transcytosis following ischemic stroke. Brain Pathology, 32(1), e13006. 34286899 10.1111/bpa.13006 PMC8713524

